# Pheromone gland transcriptome of the pink bollworm moth, *Pectinophora gossypiella*: Comparison between a laboratory and field population

**DOI:** 10.1371/journal.pone.0220187

**Published:** 2019-07-22

**Authors:** Xiaoyi Dou, Sijun Liu, Victoria Soroker, Ally Harari, Russell Jurenka

**Affiliations:** 1 Department of Entomology, Iowa State University, Ames, Iowa, United States of America; 2 The Volcani Center, Institute of Plant Protection, ARO, Bet-Dagan, Israel; Natural Resources Canada, CANADA

## Abstract

The pink bollworm, *Pectinophora gossypiella*, is a world-wide pest of cotton and in some parts of the cotton growing region is controlled by the mating disruption technique using synthetic sex pheromone. The sex pheromone consists of two compounds, (*Z*,*Z*)- and (*Z*,*E*)-7,11-hexadecadienyl acetates, in about a 50:50 ratio. However, recently, a population with sex pheromone compound ratios of about 62:38 were found in cotton fields that use mating disruption in Israel. To investigate how the change developed, we compared the pheromone gland transcriptomes between a reference laboratory population and a population obtained from an Israeli cotton field utilizing mating disruption. We analyzed four biological replicates from each population and found transcripts encoding 17 desaturases, 8 reductases, and 17 candidate acetyltransferases in both populations, which could be involved in sex pheromone biosynthesis. The expression abundance of some genes between the two populations was different. Some desaturases and candidate acetyltransferases were found to have mutated in one of the populations. The differentially expressed genes play potential roles in sex pheromone biosynthesis and could be involved in causing altered female sex pheromone ratios in the field population.

## Introduction

**Fig 1:**
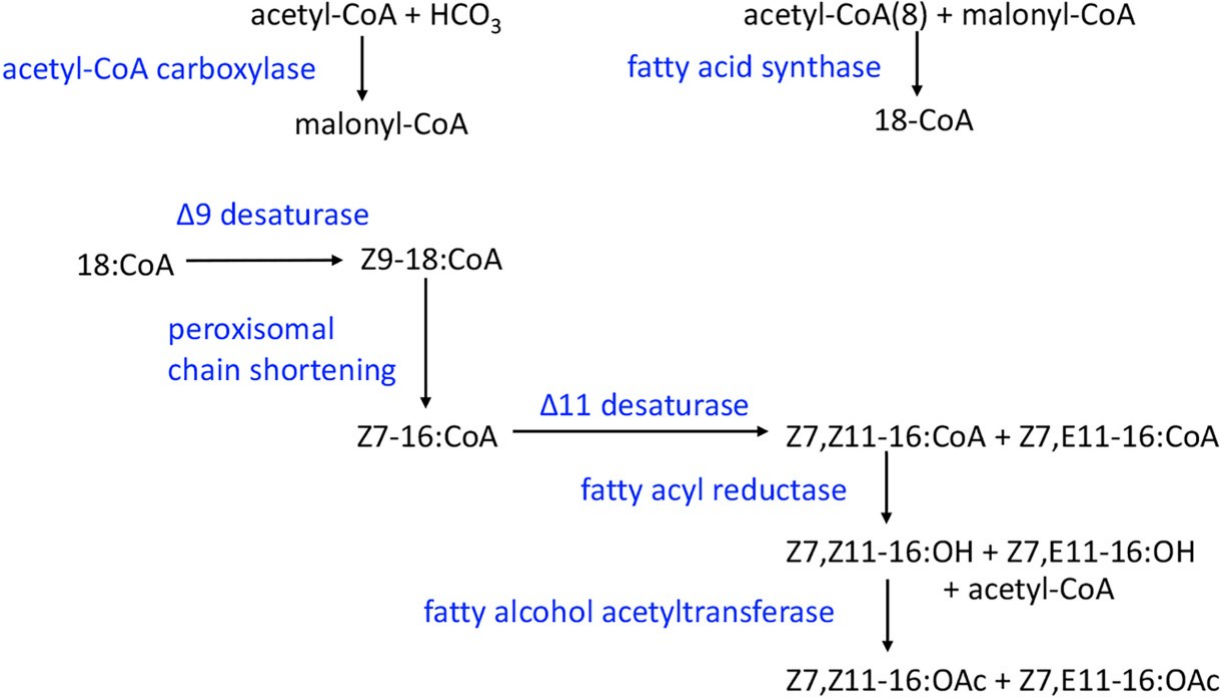
The pink bollworm sex pheromone biosynthetic pathway based on [3].

The pink bollworm (PBW), *Pectinophora gossypiella* (Lepidoptera: Gelechiidae), is a key pest of cotton in the old and new world [1]. The sex pheromone of PBW females consists of two compounds, (*Z*,*Z*)- and (*Z*,*E*)-7,11-hexadecadienyl acetates (Z,Z-7,11–16:OAc and Z,E-7,11–16:OAc) in about a 50:50 ratio [2]. The biosynthesis starts with the production of the saturated fatty-acid, stearic acid [3] (Fig 1), from the catalysis of acetyl-CoA by acetyl-CoA carboxylase (ACC) and fatty acid synthase (FAS). Then a double bond is introduced into the fatty acid chain at the Δ9 position by a Z9-desaturase to form oleic acid. After peroxisomal chain shortening by 2-carbons, another double bond is introduced by a Z11-desaturase producing both the Z and E isomers. Then the carbonyl group is modified to form a primary alcohol by fatty acyl reductase (FAR) and subsequently modified to an acetate ester by a fatty alcohol acetyltransferase (FAT). The pheromone biosynthetic pathway has been investigated using stable isotope precursors [3], but none of the genes encoding these enzymes have been identified in the PBW.

Mating disruption is an environmentally safe pest control method that has allowed growers to significantly reduce insecticide use and is now widely applied for the control of various moth pests [4–5]. Typically, mating disruption is achieved when the pheromone is released at a high dose in the active space of the pest, which negatively affects the ability of males to locate females. This technique has been applied in cotton fields all over the world including Israel and the USA as an effective control measure of the pink bollworm [6]. In Israel, practically all cotton fields are treated with sex pheromone in mating disruption, supported by use of insecticides when the pest population levels rise.

In Israel, recent repeated outbreaks in the pink bollworm population have suggested a change in population characteristics. Comparison of pheromone gland extracts of females from a recent field population outbreak to laboratory-reared females supports the possibility of a shift in sex pheromone characteristics [Harrari et al. unpublished]. Based on the sex pheromone biosynthetic pathway, we hypothesized that changes in key enzymes involved in the sex pheromone biosynthetic pathway [3] could result in changes in pheromone ratios, particularly focusing on the desaturase, reductase, and acetyltransferase enzymes (Fig 1). In this study, using the Illumina HiSeq 3000 platform, we compared the pheromone gland transcriptome between a reference laboratory population and a population obtained from cotton fields in which mating disruption has failed. We analyzed four biological replicates from each population and found transcripts encoding 17 desaturases, 8 reductases, and 17 candidate acetyltransferases. The expression abundance of some genes between the two populations was significantly different, which could be involved in causing altered female sex pheromone ratios.

## Materials and methods

### Insect collection and pheromone gland extraction and analysis

Two populations were maintained in the laboratory in Bet-Dagan, Israel as described [7]. The laboratory (Lab) population has been reared in the laboratory without any prior exposure to synthetic pheromone. The field (Field) population originated from a PBW infested cotton field near Ein Shemer, Israel (32.462472, 35.016512) in which mating disruption was utilized. Males and females were sexed in the last larval stage by a black line on the 6^th^ abdominal segment representing the developing testicles. Males and females were housed separately and newly-emerged moths were removed daily and placed into age cohort single sex cages. Adult moths were fed on a ~10% sucrose solution provided ad libitum. Pheromone glands from 3-day-old virgin females were removed 2 hours after the start of the scotophase when pheromone titers were the highest [8]. The glands were placed in hexane containing 25 ng tridecyl acetate as an internal standard and removed after twenty minutes. Gland extracts were sent to the Jurenka lab in the United States by express courier. Pheromone amounts and ratios were determined using a Hewlett Packard 5890 GC coupled to a 5972 mass selective detector. The column used to separate the extracts was a DB Wax (J&W Scientific, 30mx0.25mm). The GC was programmed at 60°C for one minute then at 5°C/min to the 230°C for 15 minutes. The mass spectrometer was set in single ion monitor mode for ions 43, 55, 67 and 81 (the 4 most abundant ions of Z,Z- and Z,E-7,11–16:OAc) and 43, 55, 61, and 69 (the 4 most abundant ions of tridecyl acetate). A 2-tailed students t-test (Microsoft Excel) was used to determine statistical significance.

### RNA isolation, cDNA library construction and Illumina sequencing

Ten pheromone glands from each population were removed from 3-day-old females during the second hour of scotophase, which is the peak period of pheromone production. Pheromone glands were immediately placed in RNAlater and frozen to -80°C and then shipped to the Jurenka lab in the United States by express courier.

Total RNA was isolated using TRIzol regent (Invitrogen, Carlsbad, CA, USA) according to the manufacturer’s protocol. The quantity of RNA was determined using the 2100 Bioanalyzer (Agilent Technologies).

One mRNA library and three stranded total RNA-Seq libraries were prepared in each population by the DNA facility of Iowa State University, Ames, Iowa, USA. The library preparations were sequenced on an Illumina HiSeq 3000 platform. The stranded total RNA-Seq libraries were sequenced with 150 pair-end and the mRNA library with 100 single reads. All sequencing reads were submitted to the SRA of NCBI under the accession number “SRP140160”.

### *De novo* assembly of short reads and gene annotation

The quality of all raw reads was checked using FastQC (Babraham Bioinformatics, UK). Low quality sequences and adaptors were removed using the Fastx-toolkit (Hannon Lab, CSHL, USA) and Trim Galore! (Felix Krueger, Babraham Bioinformatics). The *de novo* assembly was carried out using the merged reads and reads from each library respectively with the short reads assembly program Trinity [9]. After Trinity assembly, the resulting sequences were then processed using CAP3 with default parameters [10] in order to decrease the redundance of BLAST searches. The resulting clusters and singletons of more than 200 bases were locally searched against the NCBI non-redundant protein database using the BLASTx program, to obtain protein annotations of the assembled contigs. Gene Ontology terms were performed by the Blast2GO program [11] and the GO functional classification was obtained using WEGO software [12].

### Expression abundance analysis

The expression abundance of the transcripts was calculated using the method of RNA-Seq by Expectation-Maximization (RSEM) with the Trinity model. We used the RPKM (Reads Per Kilobase per Million mapped reads) value as the abundance level.

The differential expression between the two populations was measured by using the multiple test false discovery rate (FDR) calculation in the R program package ‘edgeR’ with the contigs derived from the merged reads.

### Identification of candidate genes involved in sex pheromone biosynthesis

We focused on several important genes, including acetyl-CoA carboxylase, limited β-oxidation enzymes, fatty acid synthases, desaturases, reductases and acetyltransferases. First we started by selecting the transcripts that encode these genes from the BLASTx results. Then translated these transcripts to their corresponding proteins using the TransDecoder program in Trinity. The encoding proteins were used in BLASTp to identity the genes based on homology to the NCBI non-redundant proteins database. The amino acid sequence alignment was conducted using BioEdit (http://www.mbio.ncsu.edu/bioedit/bioedit.html).

### Relative expression of several candidate genes by qPCR

One μg of total RNA was used for first-strand cDNA synthesis using ProtoScript II first strand cDNA synthesis Kit (New England BioLabs Inc.) according to the manufacturer’s protocol. The cDNAs from four replicates of each population were used as templates for qPCR. Primers are shown in S1 Table. qPCR was conducted using SYBR Green Supermix on the Applied Biosystems QuantStudio 3 (Thermo Fisher Scientific) according to the manufacturer’s protocol. The conditions of thermal cycles were: 95°C for 3 min, 40 cycles of 95°C for 15 s, 60°C for 20 s. The *P*. *gossypiella* elongation factor 1 delta was used as reference gene. The data were analyzed using the 2^-ΔΔCt^ method. A two tailed student t test in Microsoft Office Excel was used to calculate the significant difference.

### Phylogenetic analysis

Phylogenetic analysis was conducted with two genes involved in the sex pheromone biosynthetic pathway, desaturases and reductases. Here we imported 66 desaturases sequences and 70 identified reductases from other species and the genes we found in the PBW transcriptome. The amino acid sequences were aligned by the BioEdit program. The phylogenetic trees were constructed using the neighbor-joining method implemented in MEGA7 with default setting and 1000 bootstrap replicates [13].

### Statistics

The significance of differential expressed genes was calculated in R program through the FDR value of multiple comparison (https://www.R-project.org). FDRs less than 0.05 were considered as significantly differential expression between populations. Other statistical comparisons were calculated in Microsoft Office Excel using a two-tailed Student’s t-Test. Fold changes were calculated based on the RPKM value in two populations after converting to the log_2_ scale.

## Results

Analysis results of pheromone gland extracts from the Lab and Field populations are shown in Table 1. There was not a statistical difference in the amount of the ZZ and ZE isomers but there was a statistical difference in the ratio of the ZZ and ZE isomers. The Field population had a higher ZZ ratio than the Lab population.

**Table 1:** Ratios and amounts of pheromone found in glands collected from the two populations.

Population	Ratio ZZ ± SEM	Amount per gland, ng	# glands
Lab	52.5 ± 3.6	13.8 ± 6.7	17
Field	61.8 ± 3.5 *	9.6 ± 5.3	16

*Statistically significant difference *P*<0.001 2-tailed Student’s t-Test, compared to Lab population.

### Illumina sequencing and sequence assembly

Illumina sequencing of cDNA libraries prepared from the mRNA or total RNA of the pheromone glands of two PBW populations was conducted. To assess the completeness of the assembled data, the transcripts were analyzed using the BUSCO program (Benchmarking Universal Single-Copy Orthologs) using a database of arthropod genes [14] (Table 2). These results suggest that the quality of the sequencing assembly was acceptable for both populations.

**Table 2:** Analysis of sequencing results.

								BUSCO result (%)		
Population	Rep.	Library Preparation	# of raw reads	# of clean reads	%GC	# of Contigs	Ave. length	Complete (S+D)	Frag	Mis
**Lab**	1	mRNA-Seq	3.38*10^8^	2.26*10^8^	44	114125	768	97.9	1.3	0.8
	2	Stranded Total RNA-Seq	8.14*10^7^	8.07*10^7^	45	133684	707	98	1.4	0.6
	3	Stranded Total RNA-Seq	5.55*10^7^	5.48*10^7^	44	90007	800	96.8	2.1	1.1
	4	Stranded Total RNA-Seq	4.88*10^7^	4.83*10^7^	44	81495	867	97.6	1.7	0.7
**Field**	1	mRNA-Seq	3.35*10^8^	2.27*10^7^	44	155114	671	98.1	1.2	0.7
	2	Stranded Total RNA-Seq	9.91*10^7^	9.80*10^7^	44	100851	817	98.5	0.8	0.7
	3	Stranded Total RNA-Seq	6.38*10^7^	6.26*10^7^	44	86767	805	95.5	3.5	1
	4	Stranded Total RNA-Seq	4.86*10^7^	4.78*10^7^	44	82709	842	95.9	3.3	0.8
**All**						282,599	850	98	1.7	0.3

We selected the first replicate from both populations to conduct a BLASTx search with the cut-off E-value of 1.0E-5 against non-redundant protein databases in NCBI. BLASTx hits of 50,275 and 61,844 transcripts (44% and 40%) from the Lab and Field populations respectively were found. Both populations had the same top hit species. The highest hits were to *Bombyx*. *mori* (Lab: 12,424 transcripts (25%), Field: 12,863 transcripts (21%)), followed by *Danaus*. *plexippus* (9,628 hits (19%) and 10,009 hits (16%)).

All the transcripts from the two populations were annotated into different functional groups according to Gene Ontology analysis. 70,204 (61%) Lab and 82,604 (53%) Field transcripts were annotated into one or more GO categories (S1 Fig). Of the annotated transcripts, the most abundantly represented categories were “binding”, “cellular process”, “cell”, “cell part”, “metabolic process” and “catalytic activity” (more than 6,000 transcripts). In total, in Lab and Field, 27,879 and 31,534 annotated transcripts aligned to cellular component, 23,659 and 28,723 to the biological process, 18,666 and 22,347 to molecular function, respectively.

### Differential expressed gene analysis

**Fig 2:**
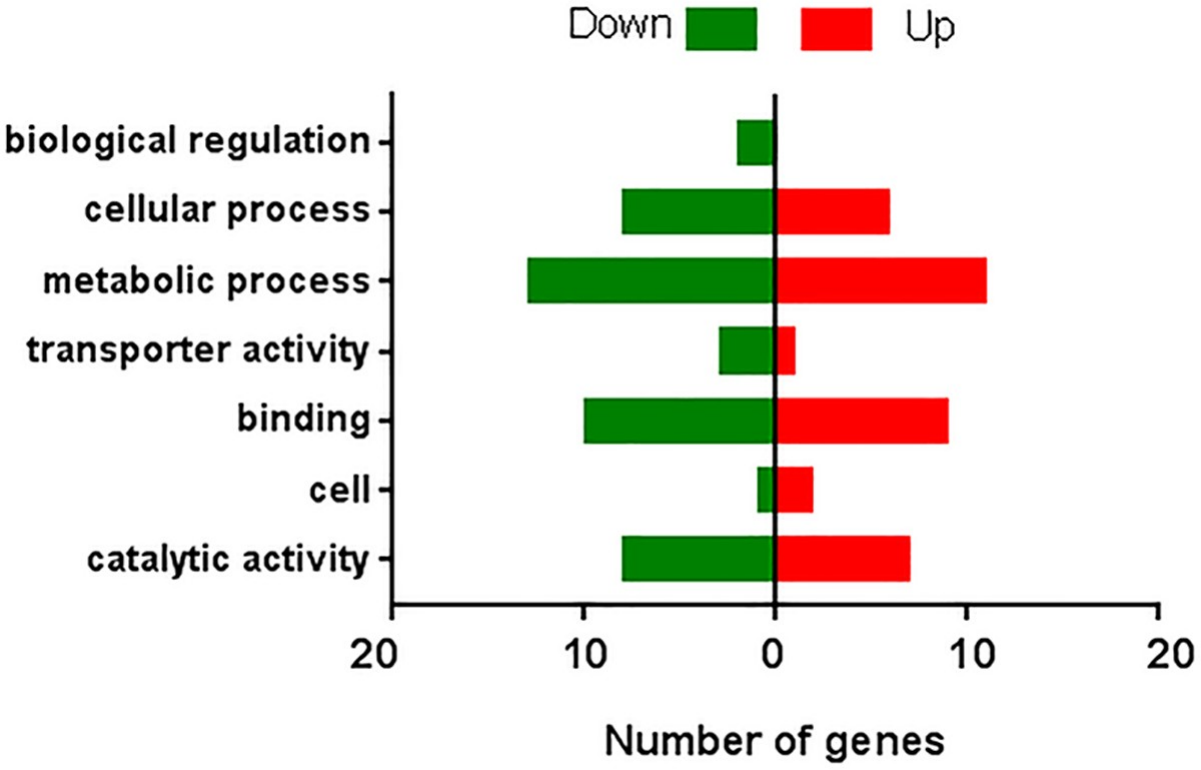
Number of differentially regulated genes in each population, grouped by gene ontology. All genes had a full length ORF with FDR less than 0.05 and logFC larger than 2 (UP) or less than -2 (DOWN). Up: Field population upregulated genes; Down: Field population downregulated genes.

We used the edgeR program to determine the differential expression analysis at the gene level using the full length contigs derived from all reads. After filtering with a log_2_ (fold change) larger than 2 or less than -2 parameter and an FDR less than 0.05, there were 88 differentially expressed genes between the Lab and Field populations (S2 Fig). 40 genes were upregulated in the Field population, while 48 were downregulated. In the upregulated genes, only 21 (52.5%) were annotated, while the remaining upregulated genes (47.5%) were unknown. In the downregulated genes, 20 genes (41.7%) were annotated while the rest (58.3%) were unknown. Number of differentially expressed genes that were classified into biological regulation, cellular process, transporter activity, etc. were similar between populations (Fig 2). The 88 differentially expressed genes are shown in S2 Table and none of the genes are directly involved in pheromone biosynthesis.

### Putative genes related to sex pheromone biosynthesis

The biosynthetic pathway (Fig 1) includes the actions of acetyl CoA carboxylase (ACC), fatty acid synthase (FAS), fatty acid desaturase (DES), β-oxidation enzymes, fatty acyl reductase (FAR), and fatty alcohol acetyltransferase (FAT). Based on BLASTx search annotation, we found members of candidate genes involved in the production of PBW sex pheromone, and then compared those genes between the two populations. In the transcriptomes, we found 1 ACC, 4 FASs, 17 DESs, 8 FARs and 17 FATs in both populations (Table 3). In addition, there were 17 transcripts in each population encoding putative β-oxidation enzymes, including 6 acyl-CoA oxidases (ACO), 2 enoyl CoA hydratases (ECH), 4 acyl-CoA dehydrogenase (ACD), 1 3-ketoacyl-CoA thiolase (3-KCT) and 4 3-hydroxyacyl CoA dehydrogenase (3-HCD) (Table 3). We also found several G-protein coupled receptors that could be involved in regulation of sex pheromone production including 2 PBANrs, 1 DHr, 1 ETHr, 4 octopamine receptors, 4 (Lab) or 5 (Field) sex peptide receptors (S4 Table). In addition, relative to pheromone transport, we found 9 chemosensory proteins and 7 odorant binding proteins (S4 Table). The comparison of amino acid sequences and abundance levels of these transcripts based on RPKM values are shown in Tables 4 and S5. However, none of them showed significant differences between populations (FDR > 0.05).

**Table 3:** Putative biosynthesis related genes in PBW pheromone glands and the first BLASTp hit in GenBank.

Transcripts	GenBank homologue Description	Accession no.*	Species	E value‡	% AA Identity
***Acetyl-CoA Carboxylase***					
ACC	Acetyl-CoA Carboxylase	XP_013176189	*Papilio xuthus*	0	89
***Fatty acid synthase***					
FAS1	fatty acid synthase-like isoform X2	XP_022831709	*Spodoptera litura*	8E-150	37
FAS2	fatty acid synthase 2	AKD01761	*Helicoverpa assulta*	0	78
FAS3	PREDICTED: fatty acid synthase	XP_013167810	*Papilio xuthus*	0	51
FAS4	fatty acid synthase-like	XP_021186732	*Helicoverpa armigera*	0	78
***Desaturase***					
DES1	acyl-CoA Delta(11) desaturase-like	XP_022825758	*Spodoptera litura*	7E-163	71
DES2	acyl-CoA delta-9 desaturase	AAK94070	*Epiphyas postvittana*	0	79
DES3	Z11-fatty acid desaturase	ALA65425	*Manduca sexta*	0	67
DES4	acyl-CoA Delta(11) desaturase-like	XP_022125992	*Pieris rapae*	1E-114	51
DES5	PREDICTED: acyl-CoA Delta(11) desaturase-like	XP_013183656	*Amyelois transitella*	0	78
DES6	E11-desaturase SFWGE11	AAQ12891	*Choristoneura parallela*	4E-151	62
DES7	sphingolipid delta(4)-desaturase DES1	XP_004930794	*Bombyx mori*	0	91
DES8	PREDICTED: acyl-CoA Delta(11) desaturase-like	XP_013195132	*Amyelois transitella*	0	94
DES9	PREDICTED: acyl-CoA Delta(11) desaturase-like	XP_011559976	*Plutella xylostella*	7E-179	70
DES10	acyl-CoA Delta(11) desaturase-like isoform X2	XP_021183601	*Helicoverpa armigera*	0	77
DES11	delta9-desaturase	AGD98721.1	*Bicyclus anynana*	2E-162	71
DES12	acyl-CoA delta-9 desaturase	CAJ27975.1	*Manduca sexta*	5E-169	91
DES13	stearoyl-CoA desaturase 5 isoform X1	XP_013192760	*Amyelois transitella*	0	78
DES14	desaturase	AIM40223	*Cydia pomonella*	0	74
DES15	acyl-CoA Delta(11) desaturase-like	XP_022125992	*Pieris rapae*	3E-119	51
DES16	acyl-CoA desaturase HassNPVE	OWR40684	*Danaus plexippus*	2E-142	66
DES 17	PREDICTED: acyl-CoA Delta(11) desaturase	XP_013193663	*Amyelois transitella*	1E-179	71
***β-oxidation enzymes***					
***Acyl-CoA oxidase***					
ACO1	PREDICTED: probable peroxisomal acyl-coenzyme A oxidase 1 isoform X2	XP_013177324	*Papilio xuthus*	1E-124	69
ACO2	peroxisomal acyl-CoA oxidase 3	AID66678	*Agrotis segetum*	0	77
ACO3	PREDICTED: probable peroxisomal acyl-coenzyme A oxidase 1	XP_013188704	*Amyelois transitella*	0	84
ACO4	PREDICTED: probable peroxisomal acyl-coenzyme A oxidase 1	XP_014366074	*Papilio machaon*	0	69
ACO5	PREDICTED: probable peroxisomal acyl-coenzyme A oxidase 1	XP_013188649	*Amyelois transitella*	3E-120	87
ACO6	peroxisomal acyl-coenzyme A oxidase 3	XP_022819471	*Spodoptera litura*	0	75
***Acyl-CoA dehdrogenase***					
ACD1	short-chain specific acyl-CoA dehydrogenase, mitochondrial-like isoform X1	XP_022830593	*Spodoptera litura*	0	75
ACD2	acyl-CoA dehydrogenase family member 9	AID66671	*Agrotis segetum*	0	67
ACD3	hypothetical protein B5V51_7750	PCG80426	*Heliothis virescens*	0	92
ACD4	very long-chain specific acyl-CoA dehydrogenase, mitochondrial isoform X1	XP_022822499	*Spodoptera litura*	0	80
***3 hydroxyacyl CoA dehydrogenase***					
3-HCD1	3-hydroxyacyl-CoA dehydrogenase type-2-like	XP_021183236	*Helicoverpa armigera*	4E-159	87
3-HCD2	3-hydroxyacyl-CoA dehydrogenase type-2	XP_021186997	*Helicoverpa armigera*	2E-160	85
3-HCD3	PREDICTED: probable 3-hydroxyacyl-CoA dehydrogenase B0272.3	XP_013190290	*Amyelois transitella*	0	90
3-HCD4	PREDICTED: probable 3-hydroxyacyl-CoA dehydrogenase B0272.3 isoform X1	XP_013140866	*Papilio polytes*	0	87
***3 ketoacyl-coa thiolase***					
3-KCT	3-ketoacyl-CoA thiolase, mitochondrial	XP_012546519	*Bombyx mori*	0	86
***Enoyl CoA hydratase***					
ECH1	enoyl-CoA hydratase domain-containing protein 3, mitochondrial isoform X1	XP_022822615	*Spodoptera litura*	1E-170	81
ECH2	PREDICTED: probable enoyl-CoA hydratase	XP_013171890	*Papilio xuthus*	3E-177	74
***Fatty-acyl reductase***					
FAR1	fatty acyl reductase	ARD71196	*Spodoptera exigua*	0	83
FAR2	putative fatty acyl-CoA reductase CG5065	XP_004925992	*Bombyx mori*	0	69
FAR3	putative fatty acyl-CoA reductase CG5065	XP_004925992	*Bombyx mori*	0	81
FAR4	putative fatty acyl-CoA reductase CG8306	XP_022824194	*Spodoptera litura*	0	80
FAR5	fatty acyl-CoA reductase wat-like	XP_021197953	*Helicoverpa armigera*	0	48
FAR6	putative fatty acyl-CoA reductase CG5065	XP_022824237	*Spodoptera litura*	0	77
FAR7	putative fatty acyl-CoA reductase CG5065	XP_022823985	*Spodoptera litura*	0	91
FAR8	fatty-acyl CoA reductase	ADI82791	*Ostrinia nubilalis*		40
***Acetyltransferase***					
FAT1	heparan-alpha-glucosaminide N-acetyltransferase	XP_004928101	*Bombyx mori*	0	79
FAT2	PREDICTED: heparan-alpha-glucosaminide N-acetyltransferase-like	XP_013191695	*Amyelois transitella*	0	78
FAT3	PREDICTED: 1-acyl-sn-glycerol-3-phosphate acyltransferase alpha-like	XP_013195392	*Amyelois transitella*	4E-168	83
FAT4	lysophospholipid acyltransferase 7-like	XP_021192342	*Helicoverpa armigera*	0	83
FAT5	PREDICTED: glycerol-3-phosphate acyltransferase 1, mitochondrial-like isoform X1	XP_013165576	*Papilio xuthus*	0	77
FAT6	lysophospholipid acyltransferase 5	XP_004933932	*Bombyx mori*	0	74
FAT7	PREDICTED: heparan-alpha-glucosaminide N-acetyltransferase-like	XP_013194974	*Amyelois transitella*	5E-132	40
FAT8	probable protein S-acyltransferase 23 isoform X1	XP_021190174	*Helicoverpa armigera*	0	98
FAT9	PREDICTED: 2-acylglycerol O-acyltransferase 2-A-like	XP_013172217	*Papilio xuthus*	0	74
FAT10	acyl-CoA:lysophosphatidylglycerol acyltransferase 1 isoform X2	XP_021181103	*Helicoverpa armigera*	0	83
FAT11	PREDICTED: glycerol-3-phosphate acyltransferase 3-like	XP_013197894	*Amyelois transitella*	0	82
FAT12	PREDICTED: sterol O-acyltransferase 2	XP_013182360	*Papilio xuthus*	0	71
FAT13	hypothetical protein B5V51_10571	PCG75995	*Heliothis virescens*	3E-141	69
FAT14	lysophospholipid acyltransferase 1 isoform X1	XP_004927037	*Bombyx mori*	0	77
FAT15	acyltransferase AGPAT3	AGG55011	*Heliothis subflexa*	3E-145	93
FAT16	PREDICTED: glycerol-3-phosphate acyltransferase 4 isoform X2	XP_013174439	*Papilio xuthus*	2E-172	82
FAT17	hypothetical protein B5V51_12259	PCG80023	*Heliothis virescens*	2E-115	91

*Accession number of the GenBank homologue.

‡E-value for the comparison of the PBW transcript AA sequence and the GenBank homologue.

**Table 4:** Comparison of candidate transcripts involved in the sex pheromone biosynthetic pathway.

	Lab population			Field population				
Gene	AA Length	Complete ORF	RPKM	AA Length	Complete ORF	RPKM	Log_2_ FC*	% AA Identity‡
*Desaturase*								
DES1	321	Y	0.5±0.1	321	Y	0.8±0.4	-0.74	100
DES2	383	Y	2362.9±488.4	351	Y	2286.3±659.2	-0.05	100
DES3	339	Y	4.9±2.8	339	Y	6.5±1.1	-0.40	100
DES4	329	Y	5.7±3.4	329	Y	8.3±2.1	-0.55	100
DES5	331	Y	0.9±0.5	331	Y	0.3±0.03	1.47	100
DES6	318	Y	300.6±54.4	318	Y	420.0±91.5	-0.48	100
DES7	321	Y	6.1±1.8	321	Y	4.7±0.9	0.37	100
DES8	327	Y	450.3±342.4	327	Y	392.8±143.7	0.20	100
DES9	331	Y	8.0±2.9	385	Y	32.6±16.4	-2.02	100
DES10	351	Y	7.6±2.6	351	Y	7.8±2.0	-0.04	100
DES11	300	Y	0.8±0.3	159	N	0.6±0.3	0.40	100
DES12	243	N	99.1±33.6	147	N	133.7±30.2	-0.43	100
DES13	372	Y	19.3±3.3	372	Y	26.2±8.5	-0.44	100
DES14	370	Y	3.9±0.9	370	Y	10.8±4.1	-1.47	100
DES15	325	Y	0.9±0.6	362	Y	0.7±0.1	0.37	100
DES16	294	Y	1.3±0.7	303	Y	2.0±1.1	-0.62	94
DES17	367	Y	5.4±1.2	367	Y	4.0±0.5	0.42	100
*Fatty-acyl reductase*								
FAR1	519	Y	2.4±0.6	519	Y	2.8±0.7	-0.23	100
FAR2	521	Y	5.3±1.8	521	Y	9.2±3.1	-0.79	100
FAR3	526	Y	9.7±2.9	552	N	9.7±0.3	0	100
FAR4	510	Y	7.2±1.8	510	Y	7.3±1.0	-0.02	100
FAR5	523	Y	19.3±10.5	523	Y	40.1±12.7	-1.06	100
FAR6	525	Y	0.9±0.3	525	Y	1.0±0.2	-0.07	100
FAR7	524	Y	0.4±0.1	524	Y	0.4±0.04	0.07	100
FAR8	451	Y	33.5±5.1	451	Y	31.6±8.8	0.09	100
*Acetyltransferase*								
FAT1	596	Y	2.1±0.5	596	Y	2.7±0.7	-0.38	100
FAT2	572	Y	11.3±2.7	586	Y	1.0±1.7	0.18	100
FAT3	270	Y	104.0±34.1	270	Y	36.5±4.8	1.51	100
FAT4	485	Y	18.5±3.7	485	Y	17.3±3.7	0.09	100
FAT5	864	Y	44.0±7.7	863	Y	45.2±5.6	-0.04	100
FAT6	480	Y	1.5±0.2	480	Y	4.6±7.0	-1.57	100
FAT7	552	Y	121.8±38.6	552	Y	42.5±9.5	1.52	100
FAT8	504	N	1.0±0.3	486	N	0.6±0.1	0.82	100
FAT9	359	Y	12.3±3.1	359	Y	9.8±0.9	0.33	100
FAT10	374	Y	2.2±0.5	378	Y	1.2±0.5	0.82	100
FAT11	497	Y	4.8±1.0	481	N	2.6±0.7	0.87	100
FAT12	469	Y	42.5±14.7	360	Y	18.7±2.5	1.12	100
FAT13	282	Y	32.3±8.6	282	Y	49.9±8.0	-0.63	100
FAT14	499	Y	24.8±1.9	499	Y	26.2±1.9	0.08	100
FAT15	245	Y	1.3±0.3	283	Y	1.8±0.3	0.51	88
FAT16	337	N	2.2±0.6	398	N	29±39.4	-3.73	100
FAT17	416	N	0.54±0	500	Y	0.2±0.1	1.17	86

* Log_2_ Fold Change (FC)—>0: up regulated in Lab population Log_2_(FC) <0: up regulated in field population

‡ % AA identity between the Lab and field populations.

#### Desaturase (DES)

Desaturases introduce double bonds into the fatty acid chain at specific positions. These desaturases were identified based on homology to other insect desaturases that have three histidine boxes with eight histidine residues that are involved in creating essential metal complexes. In PBW, at least two desaturases are involved in sex pheromone biosynthesis. A Δ9-desaturase introduces a double bond into the 18 C saturated fatty acid and after limited chain shortening, a Δ11-desaturase introduces another double bond into the chain, generating both Z,Z-7,11–16:acid and Z,E-7,11–16:acid precursors.

We found 17 transcripts encoding desaturases in Lab and Field populations. Through amino acid comparison, most of the candidate desaturases show 99% to 100% identity between the two populations. One desaturase with variations between the two populations is Lab_DES16 which had 94% identity with Field_DES16. *Lab_DES16* encodes a protein with 66% similarity with an unknown desaturase of *Danaus plexippus*. It was slightly higher expressed in the Field population (log_2_ FC = -0.62). The rest of the desaturases had 100% amino acid identity between the two populations and their abundances were at a similar levels except DES14 (log_2_ FC = -1.46) (Table 4) which was more abundant in the Field population. DES9 transcripts was also more abundant in the Field population (log_2_ FC = -2.02). However, DES5 was up regulated in the lab population with log_2_ FC = 1.47.

**Fig 3:**
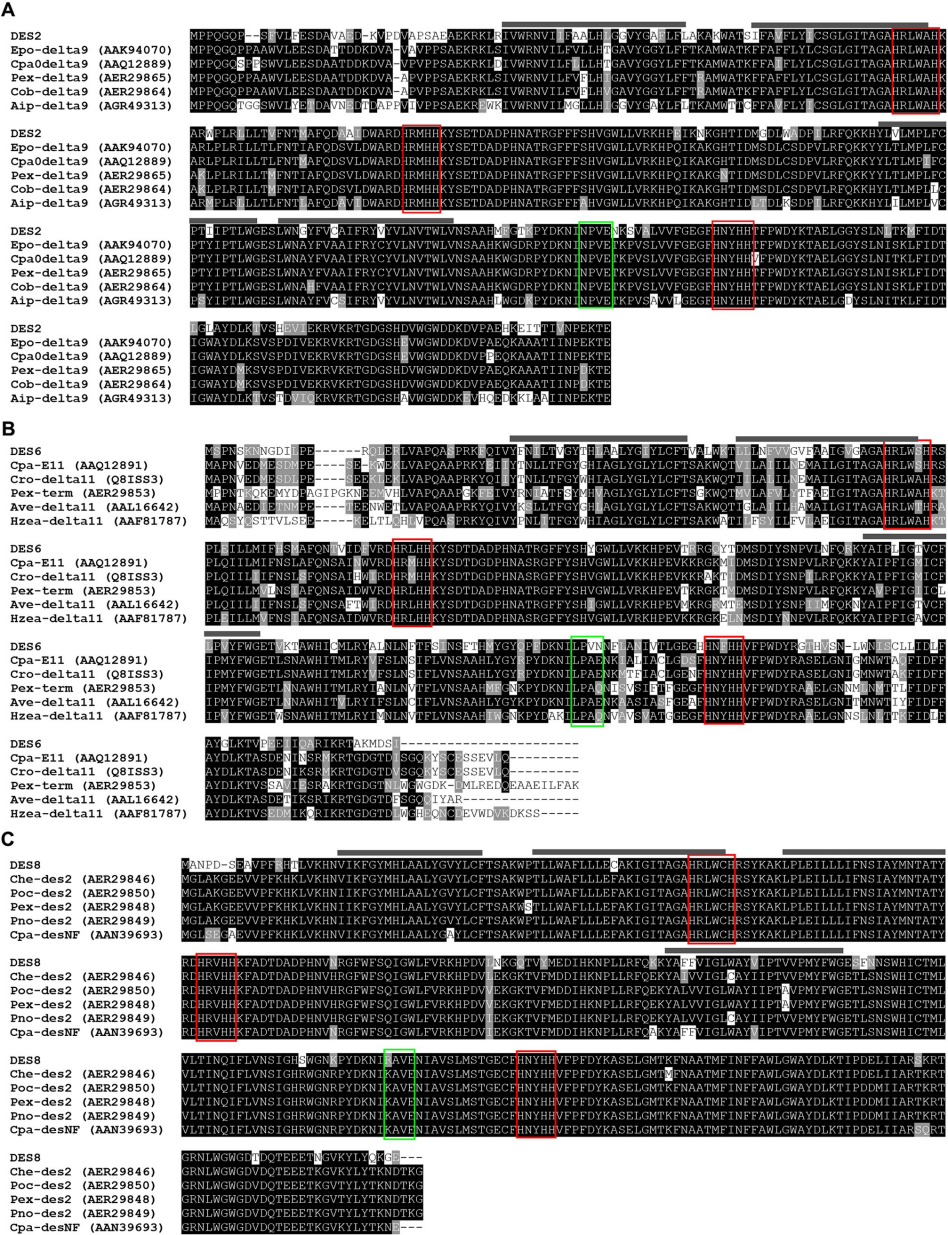
Sequence alignments of DES2, DES6 and DES8 with other desaturases from Lepidoptera. Epo: *Epiphyas postvittana*, Cpa: *Choristoneura parallela*, Pex: *Planotortrix excessana*, Cob: *Ctenopseustis obliquana*, Aip: *Agrotis ipsilon*, Cro: *Choristoneura rosaceana*, Ave: *Argyrotaenia velutinana*, Hzea: *Helicoverpa zea*, Poc: *Planotortrix octo*, Pno: *Planotortrix notophaea*. The protein ID is shown in parenthesis. The red box indicates the histidine rich motif. Gray bars above the sequence indicates transmembrane domains. The green box indicates the signature motif as described in Knipple et al. [17].

**Fig 4:**
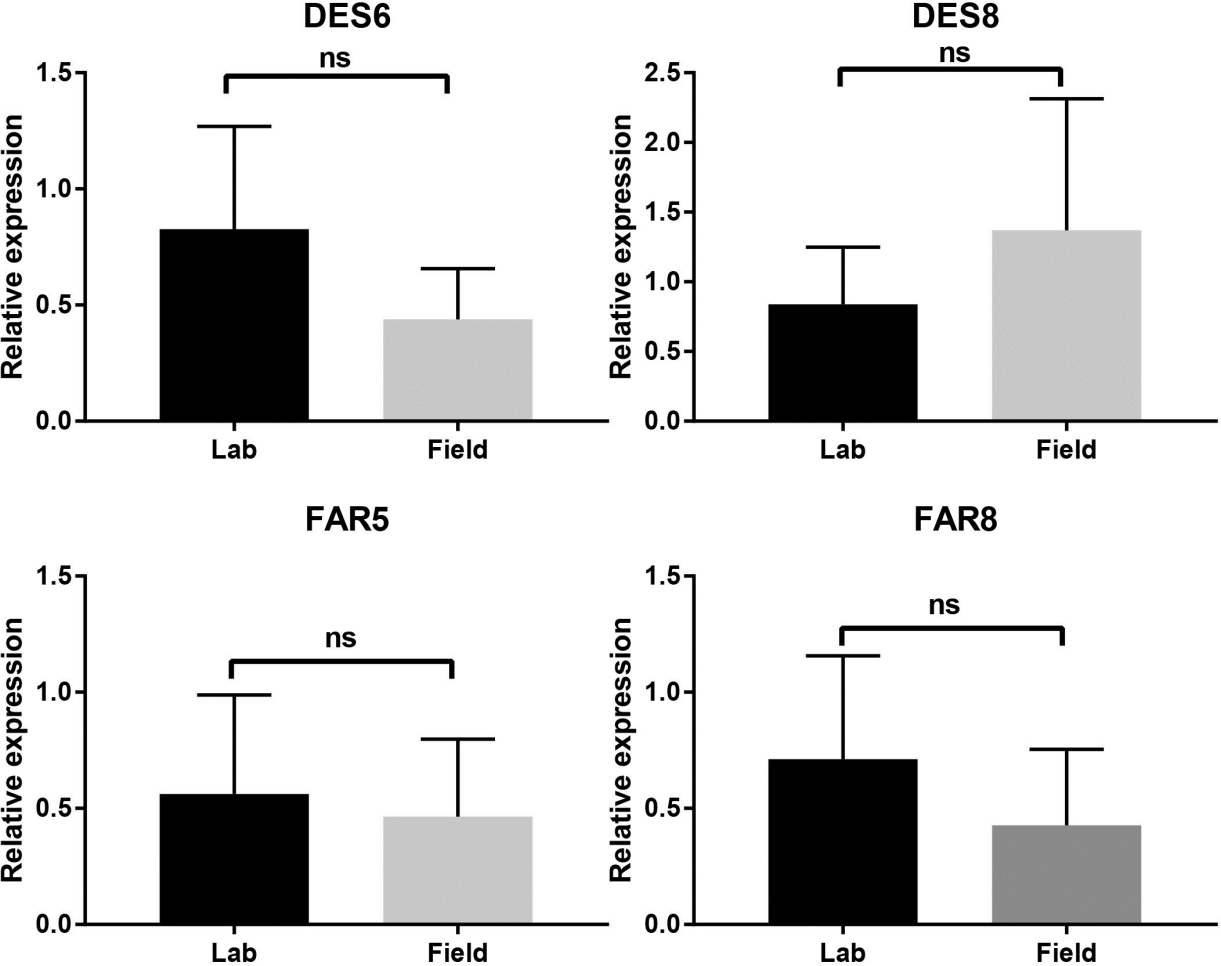
Relative expression of selected genes between Lab and Field population as determined by qPCR. ns: Non-significant, P > 0.05 (two-tailed Student’s t-Test).

The most abundant desaturase transcript is DES2 (RPKM ~ 2300) that has a 79% identity with the Δ9-desaturase of *Epiphyas postvittana*. This desaturase is probably the ubiquitous Δ9-desaturase found in other tissues and could produce oleic acid. Sequence alignment with other Lepidoptera Δ9 desaturases showed the conserved histidine rich motifs, four transmembrane domains, and the NPVE signature motif (Fig 3). The next most abundant transcripts encoding desaturases include DES6 (RPKM ~300), DES8 (RPKM ~ 400), and DES12 (RPKM ~ 100). DES6 and DES8 had 62% and 94% amino acid identity with Δ11-desaturases from the moths *Choristoneura parallela* and *Amyelois transitella*, respectively. These transcripts could encode the Δ11-desaturase in PBW to form the Z,Z-7,11–16:acid and Z,E-7,11–16:acid. The three histidine rich motifs and four transmembrane domains were found in the sequences but the specific signature motif varied in the two amino acid sequences (Fig 3). DES8 aligns with several desaturases that were found to be nonfunctional in a heterologous expression assay using yeast cells [15]. In DES8 the first histidine rich motif contains a cysteine that could potentially interfere with the di-iron binding at the catalytic site [16]. Relative expression levels by qPCR for DES6 and DES8 showed there was no significant difference between the populations (Fig 4); which is in agreement with the RPKM values not being significantly different.

**Fig 5:**
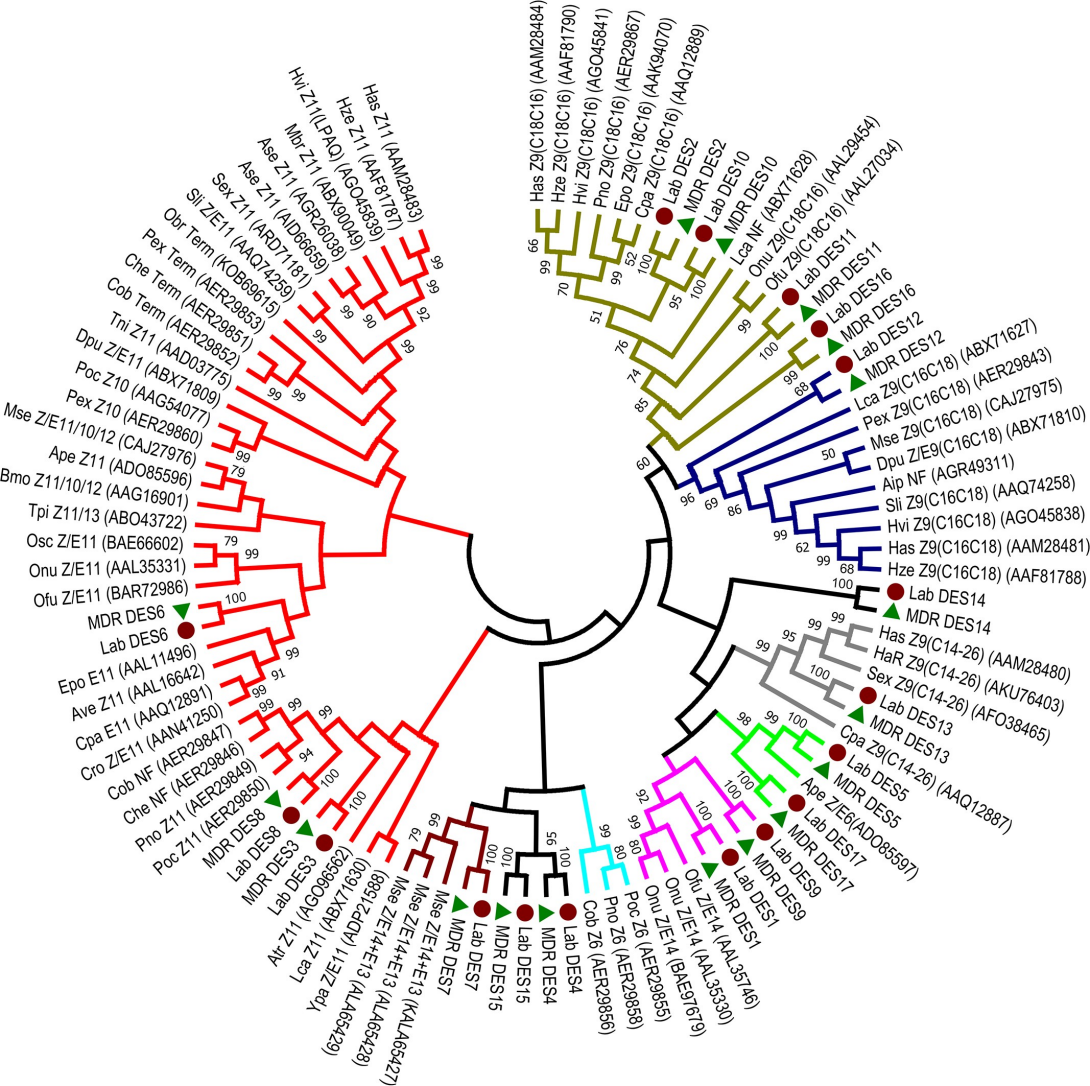
Phylogenetic relationship of DESs from Lepidoptera constructed using amino acid sequences as described in experimental methods. DESs from the Lab population were marked with a dark red circle, and from the Field population with a green triangle. Each family was classified with a different color. Clockwise from the top: brown, Δ9 desaturase (C14-26); blue, Δ9 desaturase (C16>C18); gray, Δ9 desaturase; green, Z/E Δ6 desaturase; purple, Z/E Δ14 desaturase; cyan, Z Δ6 desaturase; dark red, Z/E Δ14+ EΔ13 desaturase; Red: Δ11 desaturase.

The phylogenetic analysis of desaturases with other moth desaturases is shown in Fig 5. DES5/DES17, DES2/DES10/DES11/DES16, DES12, DES13, DES3/DES6/DES8 and DES1/ DES7/DES9 are clustered in the clades of Δ6, Δ9 (C18>C16), Δ9 (C16>C18), Δ9 (C14-26), Δ11 and Δ14, respectively. DES4, DES14 and DES15 were not similar to any class of desaturases.

#### Fatty acyl reductase (FAR)

FARs catalyze the reduction of fatty acyl-CoA to the corresponding fatty alcohol [18]. In the PBW pheromone gland transcriptome, we found 8 transcripts homologous to known insect FAR genes (Table 3). Comparison of the amino acid sequence between the two populations shows 100% identity for all 8 FARs (Table 4). We did not find the full ORF of Field_FAR3, but interestingly, the amino acid length is longer than the amino acid from the full ORF of Lab_FAR3, which indicates that Field_FAR3 covers Lab_FAR3. Among these transcripts, four encode proteins that have 77% - 91% identity with fatty acyl reductases of *Spodoptera exigua*. The rest have various similarities with *Bombyx mori*, *Helicoverpa armigera* and *Ostrinia nubilalis*. FAR5 and FAR8 had highest abundance in PBW PG (RPKM ~ 30), while FAR5 was expressed higher in the Field population (log_2_ FC = -1.06). Relative expressions were checked by qPCR for these two transcripts, both were not significantly different (Fig 4). The RPKM values were also not significantly different. Other FARs had a low abundance with RPKM values of less than 10 and with 100% AA identity (Table 3).

**Fig 6:**
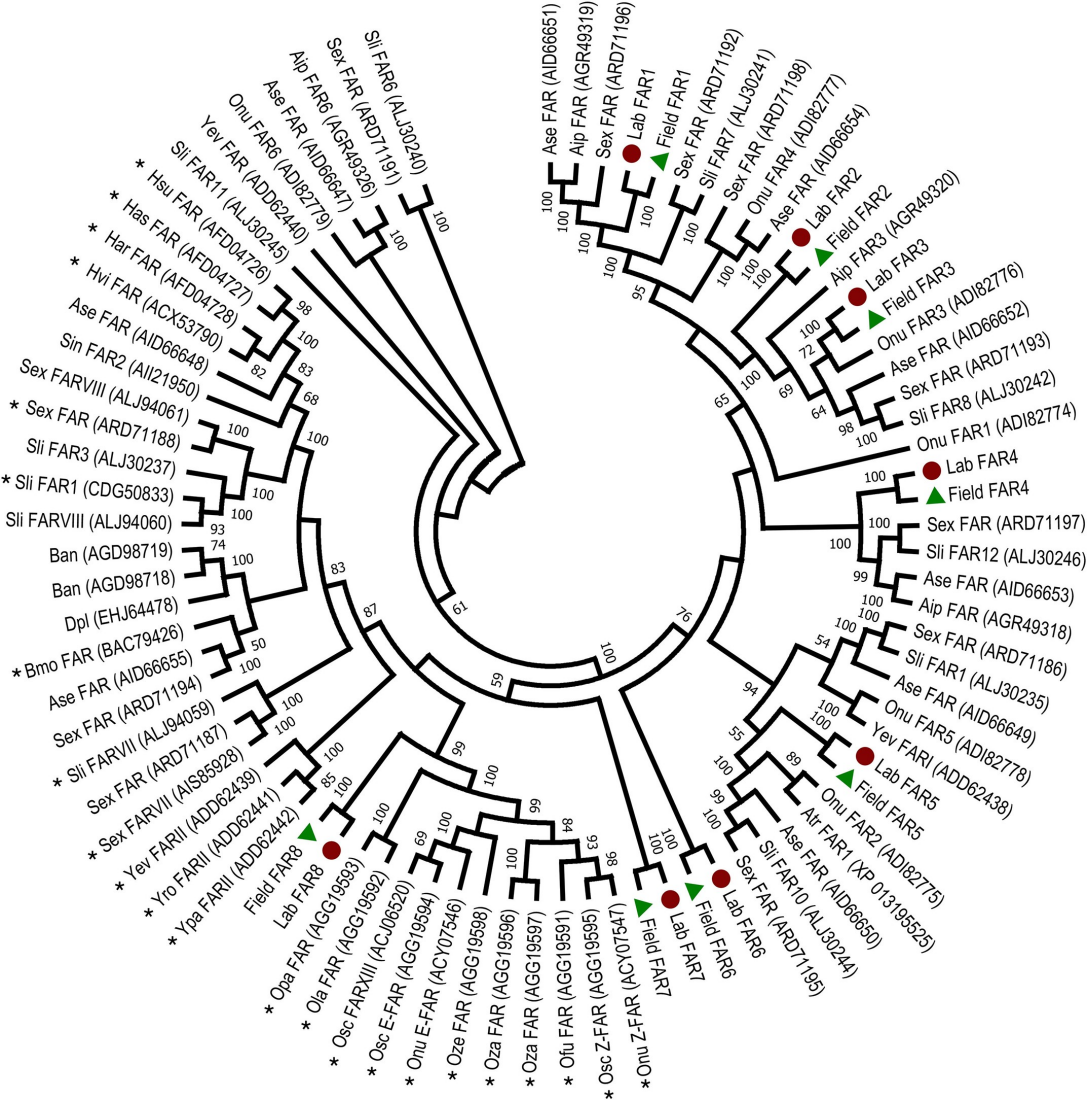
Phylogenetic relationship of FARs from Lepidoptera constructed using amino acid sequences as described in Materials and Methods. FARs from the Lab population were marked with a dark red circle, and from the Field population with a green triangle. FARs specifically biochemically identified from pheromone glands are marked with an asterisk.

Based on the phylogenetic analysis of moth FARs (Fig 6), two candidate FARs were identified to be likely involved in pheromone biosynthesis, FAR7 and FAR8. FAR7 forms a clade with the identified FARs found in pheromone glands of *Bombyx mori* [18], *Helicoverpa* [19], *Spodoptera littoralis* [20], and *Yponomeuta* [21]. FAR8 is in the clade of *Ostrinia* [22] reductases.

#### Fatty acetyltransferases (FAT)

Fatty acetyltransferases convert fatty alcohols to acetate esters. This gene has not been identified in any insects at the molecular level [23]. In the PBW PG transcriptome, 17 candidate FATs were found (Table 3) based on BLASTp search results and further filtered to include only potential transferases that have transmembrane domains using the prediction programs TMHMM Server and TMpred Server. Some of the transcripts hit to N- or S- acetyltransferases from other moths, but the biochemical function for these acetyltransferases has not been confirmed so we included them here. We found 14 and 15 full sequences in the Lab and Field populations, respectively. All genes were 100% identical between the two populations, except for FAT15 and FAT17 (Table 4). The highest identity of Lab_FAT15 to Field transcripts is Field_FAT15, which shows 88% similarity. Lab_FAT15 is homologous to the acyltransferase AGPAT3 of *H*. *subflexa* with 93% identity, while Field_FAT15 showed 91% similarity to the AGPAT alpha-like of *S*. *litura* (Gene Bank: PCG64070). Lab_FAT17, as the partial sequence, showed 86% similarity with Field_FAT17. Lab_FAT17 was homologous to the predicted protein of *H*. *virescens* (Gene bank: PCG80023) with 91% identity, while Field_FAT17 showed 87% identity to the same entry. These two genes had low abundance levels (RPKM <1). The most abundant genes were *FAT3* and *FAT7* with the log2 Fold changes larger than 1.5, indicating lower expression in the Field population. Also, the abundance levels of FAT8, FAT10, FAT11, FAT17 in the Field population were lower (Table 4), while FAT6 and FAT16 had higher abundance in the Field population (log_2_ FC = -1.57, -3.73).

See S1 File for results and discussion of transcripts involved in saturated fatty acid biosynthesis, β-oxidation, G-protein coupled receptors, and carrier proteins.

## Discussion

The pink bollworm as a world-wide pest, has been well controlled by the mating disruption technique using artificial sex pheromone in Israel until recently when repeated outbreaks have been documented (Harari et al., unpublished). We have found that the females from the Field populations are producing a higher ratio of the ZZ isomer (Table 1) and that males can find these females when exposed to mating disruption pheromone (Harari et al. unpublished). Therefore to understand how females could shift the ratio of sex pheromone components we undertook a transcriptome study to identify the genes that are involved in the sex pheromone biosynthetic pathway and transport in the pheromone gland, similar to studies in other moths [24–30].

By sequencing the PBW pheromone gland transcriptome of Lab and Field populations, we found 64 transcripts encoding enzymes that are putatively involved in pheromone biosynthesis. This is the first study reporting the key enzymes involved in PBW sex pheromone biosynthesis along with a comparison between Lab and Field populations. Although most transcripts were identical between populations there were some differences in transcripts encoding desaturases and acetyltransferases. For desaturases, Lab_DES16 had a 94% identity to Field_DES16 in all four replications. *DES16* is likely encoding a Δ9 desaturase and their abundance level based on RPKM values is very low indicating that this desaturase is probably not involved in pheromone biosynthesis. The most abundant desaturase transcripts were *DES2*, *DES6* and *DES8*, which may encode Δ9 and Δ11 desaturases, respectively, because their sequences are similar to identified desaturase sequences from other moth pheromone glands [15–16, 31]. However DES8 aligns with other desaturases that were found to be nonfunctional as a desaturase in the yeast expression system [15]. DES6 is expressed at a lower level in the Lab population, indicating the possible role of this Δ11 in changing sex pheromone ratio, although a functional analysis needs to be conducted to confirm the type of desaturase encoded by each transcript.

The Z and E Δ11 isomers could be produced by one desaturase or by separate desaturases. *DES6* was the only desaturase that had relatively high transcript abundance and aligned with other functionally described Δ11 desaturases indicating that this desaturase could produce both the E and Z Δ11 isomers. Some Δ11 desaturases have been shown to produce only a Z or E isomer [15–16, 32–33] and some desaturases are bifunctional and could produce both isomers [31–33]. It has been demonstrated that a single amino acid substitution could switch a Δ11-desaturase that produces primarily Z isomer to one that produces mostly the E isomer [16]. We did not find single amino acid substitutions in the comparison of the highly expressed desaturases so other mechanisms must account for the change in the pheromone ratios we have observed in the Field population.

We found two FATs that differed in amino acid sequence between the two populations. *FAT15* and *FAT17* had 88% and 86% identity between the two populations. However, the RPKM values were very low in both populations indicating that they may not be involved in pheromone biosynthesis. The most abundant FATs were *FAT3* and *FAT7* in the Lab population but were statistically lower in the Field population. In the Field population there were 4 FATs (FAT3, FAT5, FAT7, FAT13) that were the most abundant transcripts with RPKM between 30 and 50. These are all putative FATs because none have been identified at the molecular level in moth pheromone glands as the actual transferase that produces acetate esters.

The other major enzyme involved in pheromone production is the FAR that produces the alcohol required for the FAT to make acetate esters. We found 8 FARs in PBW pheromone glands with no sequence differences found between the two populations. The two most abundant transcripts were FAR5 and FAR8 for both populations with a significant difference in FAR5 that was higher in the Field population. Either of these could be involved in producing the alcohols required for acetate ester production since they are related to FARs identified from other moth pheromone glands [18–22].

Moreover, we found 9 CSPs and 7 OBPs from both populations in all 4 replications that had 100% identity and their expression level was also similar (S4 Table). These proteins could be involved in the transport of pheromone components to the surface of the pheromone gland for release. However we did not find any differences in the transcripts encoding these proteins indicating that the increased ratio of ZZ isomers in the Field populations may not be caused by the differential transportation of sex pheromone. In addition, in the analysis of differential expressed genes, we found many genes with an unknown annotation (S2 Table). These unknown genes with differential expression may play roles in changing the sex pheromone ratio.

The increase in the ZZ isomer that we have found in the field population could be due to variation in abundance of key enzyme(s) found in the biosynthetic pathway. Desaturase abundance with an increase in the Δ11 desaturase that produces the Z isomer is possible. Variation in putative FATs were found between the two populations and one or more of these could be involved in preferentially acetylating the ZZ isomer. Tortricid moths utilize a FAT that prefers the Z isomer of Δ11 fatty alcohols [34] and a similar FAT could be involved in producing the increased ZZ isomer in the field PBW. An increase in the abundance of one of the FARs that forms the ZZ alcohol could also be involved in increasing the ZZ isomer. A combination of enzymes that favor the ZZ isomer could also account for the increase in this isomer in the field population.

Another interesting finding is that the relative expression level of enzymes involved in pheromone biosynthesis are not similar among all enzymes in the biosynthetic pathway. The relative expression levels apparently decline in enzymes as they occur later in the biosynthetic pathway. The desaturases are expressed at the highest level whereas the next enzyme in the pathway, the fatty-acid reductase is expressed at a lower level. This phenomenon has also been found in transcriptome studies of other moth pheromone glands [25–30, 34–38]. The next enzymes in the pathway are either an alcohol dehydrogenase in those moths that produce aldehydes or an acetyltransferase in those moths that produce acetate esters; neither of which has been identified at the molecular level. If these enzymes are expressed at relatively low levels it becomes difficult to predict which transcript encodes these enzymes [25].

This comparative transcriptome study has provided sequence and transcript abundance information that can be used to identify key enzymes involved in pheromone biosynthesis in the PBW moth. Identification of the enzyme will require expressing each enzyme in an heterologous expression system followed by a functional assay. Identifying factors in the field population that are involved in the increased ZZ isomer ratios will require further research. Other factors in addition to enzyme changes could be selective degradation of pheromone, the regulation of releasing pheromone, or some abiotic environmental factors.

## Supporting information

S1 Fig Gene Ontology classification and comparison of PBW transcripts of two populations. (TIF)

S2 Fig Volcano plot for differential expressed genes between Lab and Field populations. FC: Fold Change. FDR: False discovery rate. Black dots: FDR>0.05. Not significant; Red dots: FDR < 0.05, Significant.
(TIF)

S1 Table Primers for qPCR. (PDF)

S2 Table 88 differentially expressed genes between populations. (PDF)

S3 Table Comparison of candidate transcripts involved in saturated fatty acid biosynthesis and β-oxidation. (PDF)

S4 Table Putative G-protein coupled receptors, odorant binding proteins, chemosensory proteins in PBW pheromone glands and the first BLASTp hit in GenBank. (PDF)

S5 Table Comparison of candidate transcripts of G-protein coupled receptors, odorant binding proteins, chemosensory proteins in PBW pheromone glands. (PDF)

S1 File Transcripts involved in saturated fatty acid biosynthesis, β-oxidation, G-protein coupled receptors, and carrier proteins. (PDF)

S2 File All the amino acid sequences we found in this study. (PDF)
