# Supplementary figures and images for: Pheromone gland transcriptome of the pink bollworm moth, Pectinophora gossypiella: Comparison between a laboratory and field population
Source: PLoS One. 2019 Jul 22;14(7):e0220187. doi: 10.1371/journal.pone.0220187 (PMC6645563; doi:10.1371/journal.pone.0220187)

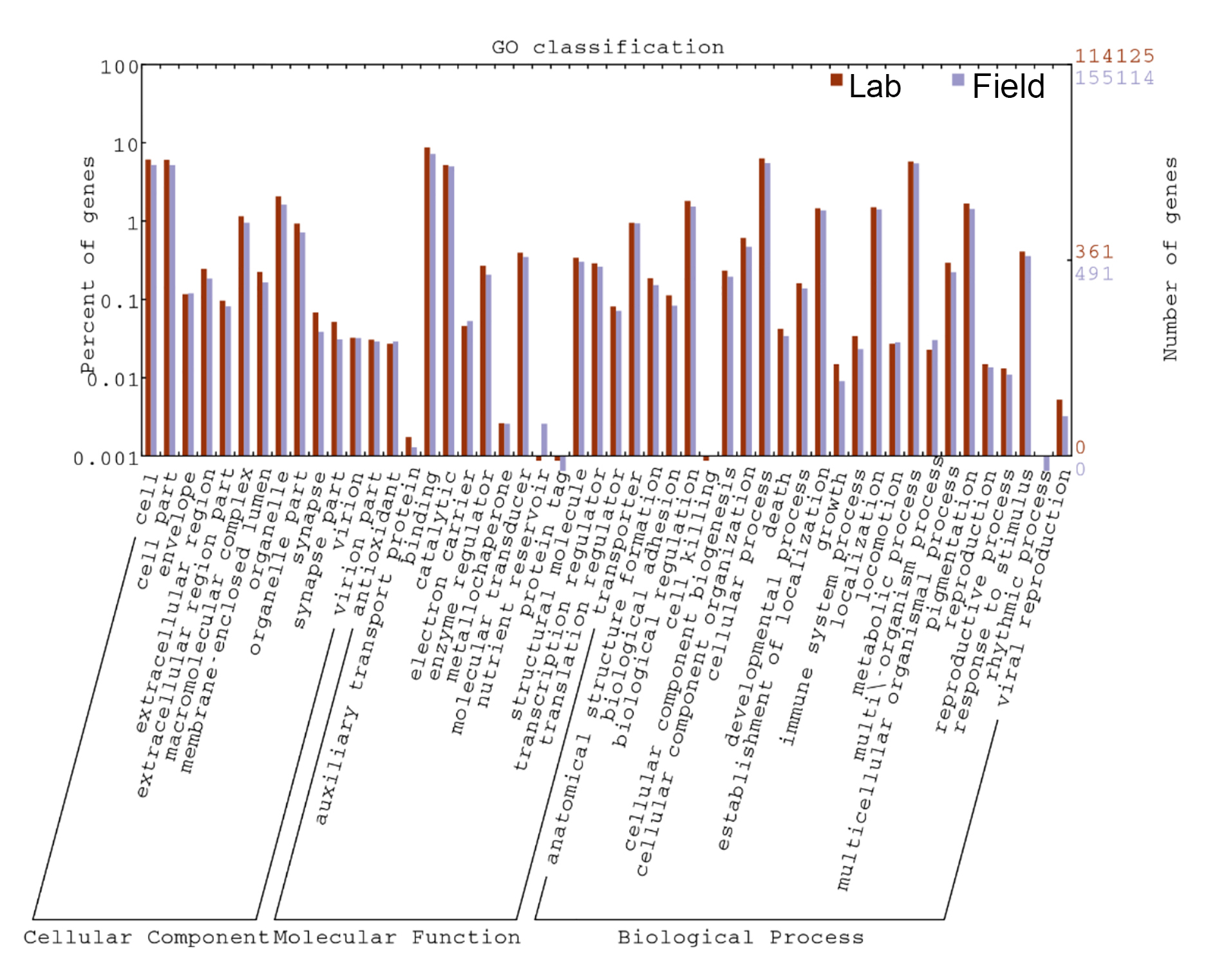

Supplement: S1 Fig — (TIF) [file pone.0220187.s001.tif]

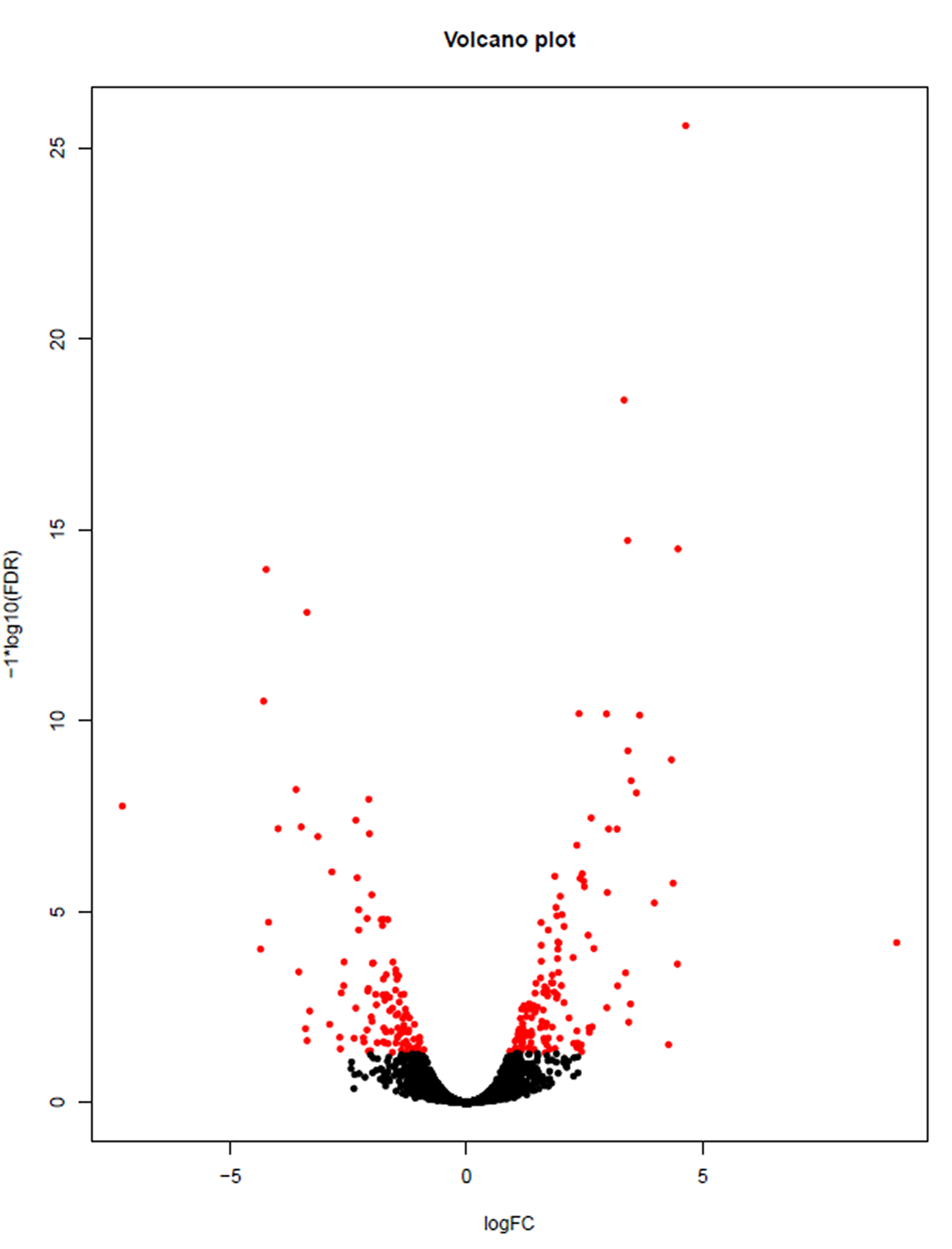

Supplement: S2 Fig — FC: Fold Change. FDR: False discovery rate. Black dots: FDR>0.05. Not significant; Red dots: FDR < 0.05, Significant. (TIF) [file pone.0220187.s002.tif]
