# Supplementary material for: Pheromone gland transcriptome of the pink bollworm moth, Pectinophora gossypiella: Comparison between a laboratory and field population
Source: PLoS One. 2019 Jul 22;14(7):e0220187. doi: 10.1371/journal.pone.0220187 (PMC6645563; doi:10.1371/journal.pone.0220187)
Supplement: S1 Table — (PDF) [file pone.0220187.s003.pdf]

S1 Table. Primers for qPCR

DES6	f: 5’-CAGACGCCGATCCTCATAAC-3’	r: 5’-CACACTGTTCCAATTAGCGG-3’
DES8	f: 5’-TCCTGACGTCCTCAACAAAG-3’	r: 5’-AAGTACATCGGCACGACCGT-3’
FAR5	f: 5’-GTGCACGGAACACAGCAAA-3’	r: 5’-CACAGTCTCAGCCATCTGGA-3’
FAR8	f: 5’-ACTACGTCTGTGCAATCCGG-3’	r: 5’-GGTCGGACTACAGCAACTG-3’
elongation factor 1 delta	f: 5’-TTGGAGGCACCCCACAGAAG-3’	r: 5’-CCTGCCCTTATTGCCAAGTC-3’
