# Supplementary material for: Pheromone gland transcriptome of the pink bollworm moth, Pectinophora gossypiella: Comparison between a laboratory and field population
Source: PLoS One. 2019 Jul 22;14(7):e0220187. doi: 10.1371/journal.pone.0220187 (PMC6645563; doi:10.1371/journal.pone.0220187)
Supplement: S2 Table — (PDF) [file pone.0220187.s004.pdf]

Table S2. The 88 differentially expressed genes between Lab and Field populations.

Gene description	AA length	Log2FC	P Value	FDR
**Field Upregulated**				
facilitated trehalose transporter Tret1-like	526	4.243	3.26E-17	1.07E-14
Unknown	104	3.379	5.03E-16	1.42E-13
Unknown	661	4.298	1.28E-13	3.01E-11
Unknown	709	3.611	3.91E-11	6.21E-09
trypsin 5G1-like	258	2.071	7.75E-11	1.12E-08
Unknown	195	7.290	1.21E-10	1.69E-08
digestive cysteine proteinase 1-like	549	2.345	3.09E-10	3.95E-08
laccase-1-like	592	3.503	4.80E-10	5.94E-08
Unknown	108	3.990	5.36E-10	6.59E-08
Unknown	337	2.056	7.46E-10	8.92E-08
Unknown	330	3.147	9.16E-10	1.06E-07
Unknown	110	2.851	8.97E-09	8.93E-07
serine protease 24	512	2.315	1.31E-08	1.27E-06
Unknown	1680	2.008	4.07E-08	3.55E-06
Insect cuticle protein	153	2.284	1.08E-07	8.80E-06
regucalcin-like	339	2.107	1.87E-07	1.48E-05
protein-L-isoaspartate(D-aspartate) O-methyltransferase	300	4.194	2.44E-07	1.86E-05
Unknown	311	2.283	4.01E-07	2.97E-05
ysozyme-like	161	4.363	1.39E-06	9.41E-05
cytochrome P450 4C1-like	507	2.593	3.35E-06	0.000204
cuticle protein 16.5, isoform B-like isoform X1	314	3.552	6.52E-06	0.000371
aldo/keto reductase	112	2.604	1.76E-05	0.000857
60S ribosomal protein L9	195	2.079	2.16E-05	0.001014
Unknown	539	2.091	2.55E-05	0.001178
Unknown	116	2.652	2.92E-05	0.001321
Unknown	126	2.346	8.56E-05	0.003283
Unknown	983	3.321	0.000106	0.003939
gamma-interferon-inducible lysosomal thiol reductase-	238	2.018	0.000165	0.005629
Unknown	164	2.002	0.000227	0.00737
PH, RCC1 and FYVE domains-containing protein 1	373	2.899	0.000282	0.008783
attacin-like	155	3.411	0.000389	0.011385
RNA-directed DNA polymerase from mobile element	497	2.109	0.000428	0.012351
Unknown	128	2.684	0.000748	0.019236
PREDICTED: venom peptide BmKAPI-like	138	2.184	0.000776	0.019773
phospholipid scramblase 1-like	222	2.381	0.000805	0.020281
gloverin-like	194	3.377	0.000972	0.023594
Unknown	775	2.173	0.00108	0.02556
Unknown	170	2.674	0.001824	0.038556
Unknown	1407	2.037	0.00211	0.043083
cytochrome P450 6B2-like	356	2.077	0.002191	0.04434
**Field Downregulated**				
Unknown	838	-4.640	3.47E-29	2.54E-26
gag-pol polyprotein	952	-3.337	8.05E-22	3.89E-19
Unknown	978	-3.411	5.35E-18	1.87E-15
Unknown	387	-4.476	9.07E-18	3.10E-15
Unknown	187	-2.381	2.87E-13	6.40E-11

Unknown	905	-2.964	2.92E-13	6.47E-11
Unknown	1312	-3.666	3.23E-13	7.05E-11
Unknown	115	-3.416	3.16E-12	6.01E-10
hypodermin-A-like isoform X2	277	-4.339	5.60E-12	1.03E-09
olfactory receptor 16	354	-3.484	2.14E-11	3.64E-09
glutathione S-transferase siama 2	205	-3.598	4.96E-11	7.58E-09
Unknown	898	-2.639	2.66E-10	3.43E-08
Unknown	152	-3.008	5.46E-10	6.68E-08
collagen alpha-1(XV) chain-like	128	-3.186	5.52E-10	6.73E-08
Unknown	219	-2.337	1.62E-09	1.79E-07
Unknown	117	-2.449	1.01E-08	1.00E-06
synaptic vesicle glycoprotein 2C-like	530	-2.407	1.37E-08	1.32E-06
Unknown	182	-2.480	1.65E-08	1.56E-06
Unknown	133	-4.374	1.91E-08	1.77E-06
mucin-19-like isoform X1	391	-2.494	2.38E-08	2.17E-06
Unknown	1761	-2.980	3.49E-08	3.10E-06
Unknown	410	-3.976	6.88E-08	5.77E-06
Beta-ureidopropionase	417	-2.020	1.48E-07	1.18E-05
zinc carboxypeptidase-like	218	-2.065	3.17E-07	2.39E-05
Unknown	564	-2.575	5.64E-07	4.08E-05
Unknown	291	-9.109	9.00E-07	6.35E-05
piggyBac transposable element-derived protein 4-like	274	-2.697	1.32E-06	9.04E-05
Unknown	1052	-2.255	2.44E-06	0.000156
Unknown	678	-4.467	3.85E-06	0.000231
Unknown	117	-3.368	7.04E-06	0.000395
cytochrome P450 4c3-like	499	-2.009	1.75E-05	0.000852
glutathione S-transferase epsilon 8	235	-3.199	1.78E-05	0.000862
facilitated trehalose transporter Tret1-like	488	-2.064	5.75E-05	0.002376
probable cytochrome P450 6a23	609	-3.472	6.37E-05	0.002582
Unknown	621	-2.972	8.35E-05	0.003227
trichohyalin isoform X1	297	-2.170	0.000177	0.005958
Unknown	104	-3.435	0.00024	0.007695
Unknown	738	-2.658	0.000338	0.010195
DNA ligase	408	-2.593	0.000364	0.010832
Unknown	192	-2.338	0.000462	0.013103
Unknown	965	-2.603	0.000522	0.014284
calexcitin-2-like	201	-2.351	0.001155	0.026934
synaptic vesicle glycoprotein 2B-like	512	-2.269	0.0012	0.027804
Unknown	107	-4.277	0.001307	0.029929
Unknown	152	-2.428	0.001318	0.03009
Unknown	844	-2.376	0.001532	0.033786
15-hydroxyprostaglandin dehydrogenase NAD(+)]-like	273	-2.339	0.001635	0.035712
monocarboxylate transporter 12-like	521	-2.438	0.002265	0.045546
