# Supplementary material for: Pheromone gland transcriptome of the pink bollworm moth, Pectinophora gossypiella: Comparison between a laboratory and field population
Source: PLoS One. 2019 Jul 22;14(7):e0220187. doi: 10.1371/journal.pone.0220187 (PMC6645563; doi:10.1371/journal.pone.0220187)
Supplement: S3 Table — (PDF) [file pone.0220187.s005.pdf]

Table S3 Comparison of candidate transcripts involved in saturated fatty acid biosynthesis and β-oxidation.

Gene	Lab population			Field population			Log2 Fold Change	% Identity*
	AA Length	Complete ORF	RPKM	AA Length	Complete ORF	RPKM		
Acetyl-CoA Carboxylase								
ACC	2394	Y	13.2±4.9	2394	Y	10.0±4.4	0.40	100
Fatty acid synthase								
FAS1	717	N	1.8±1.2	1531	N	1.6±0.5	1.12	100
FAS2	1348	N	28.9±15.6	1368	N	14.0±2.0	2.05	100
FAS3	985	N	10.6±7.8	1611	N	7.0±2.3	1.50	100
FAS4	1050	N	84.4±11.4	489	N	38.3±23.4	2.20	100
β-oxidation enzymes								
Acyl-CoA oxidase								
ACO1	251	N	13.6±1.2	244	N	13.5±2.0	0.02	100
ACO2	695	Y	3.8±0.9	695	Y	3.6±0.1	0.10	100
ACO3	668	Y	25.1±7.2	668	Y	22.4±2.0	0.16	100
ACO4	444	N	3.7±0.8	447	N	10.1±1.9	-1.46	100
ACO5	191	N	0.3	575	N	0.6±0.2	-1.17	100
ACO6	686	Y	0.6±0.2	686	Y	0.6±0.1	-0.04	100
Acyl-CoA dehdrogenase								
ACD1	409	Y	4.8±3.2	409	Y	4.0±1.8	0.26	100
ACD2	608	Y	7.7±1.9	608	Y	7.0±0.9	0.13	100
ACD3	407	Y	11.1±2.4	407	Y	14.5±2.1	0.38	100
ACD4	577	Y	29.5±9.0	577	Y	31.2±7.7	-0.08	100
3_hydroxyacyl_CoA_dehydrogenase								
3-HCD1	256	Y	4.1±1.9	256	Y	4.0±1.5	0. 04	100
3-HCD2	255	Y	11.1±2.6	255	Y	6.0±1.1	0.89	100
3-HCD3	312	Y	35.2±18.8	309	Y	26.2±11.5	0.43	100
3-HCD4	307	Y	8.6±1.5	307	Y	6.5±1.8	0.41	100
3_ketoacyl-CoA_thiolase								
3-KCT	400	Y	4.0±1.8	400	Y	2.9±0.3	0.44	100
enoyl_CoA_hydratase								
ECH1	278	Y	2.3±0.5	278	Y	1.7±0.3	0.44	100
ECH2	332	Y	12.7±4.8	332	Y	10.1±2.2	0.33	100

* % identity between the Lab and Field populations.
