# Supplementary material for: Pheromone gland transcriptome of the pink bollworm moth, Pectinophora gossypiella: Comparison between a laboratory and field population
Source: PLoS One. 2019 Jul 22;14(7):e0220187. doi: 10.1371/journal.pone.0220187 (PMC6645563; doi:10.1371/journal.pone.0220187)
Supplement: S4 Table — (PDF) [file pone.0220187.s006.pdf]

Table S4. Putative G-protein coupled receptors, odorant binding proteins, chemosensory proteins in PBW pheromone glands and the first BLASTp hit in GenBank.

Transcripts	GenBank homologue description	Accession no.*	Species	E value‡	Identity (%)
**Diapause hormone receptor**					
DHr	diapause hormone receptor	ANB78221	*Helicoverpa armigera*	0	66
**Ecdysis-triggering hormone receptor**					
ETHr	Ecdysis triggering hormone receptor subtype-A	OWR50706	*Danaus plexippus*	0	74
**Octopamine receptor**					
Octo_r1	octopamine receptor beta-2R-like	XP_013165403	*Papilio xuthus*	0	86
Octo_r2	octopamine receptor Oamb isoform X2	XP_022827336	*Spodoptera litura*	0	92
**Sex peptide receptor**					
SPR1	sex peptide receptor isoform X1	XP_013178706	*Papilio xuthus*	0	85
SPR2	PREDICTED: sex peptide receptor-like	XP_013187671	*Amyelois transitella*	0	84
SPR3	sex peptide receptor-like	XP_013195850	*Amyelois transitella*	0	94
SPR4	sex peptide receptor-like isoform X2	XP_022817535	*Spodoptera litura*	0	82
Field_SPR5	PREDICTED: sex peptide receptor-like	XP_013187496	*Amyelois transitella*	0	79
**PBAN receptor**					
PBANr1	pyrokinin-1 receptor-like isoform X2	XP_022814705	*Spodoptera litura*	0	83
PBANr2	pyrokinin-1 receptor-like isoform X1	XP_022814703	*Spodoptera litura*	0	83
**Chemosensory proteins**					
CSP1	chemosensory protein 16	BAV56820	*Ostrinia furnacalis*	1E-20	44
CSP2	chemosensory protein	AII01028	*Dendrolimus kikuchii*	5E-71	81
CSP3	chemosensory protein 7	BAV56811	*Ostrinia furnacalis*	6E-65	72
CSP4	putative chemosensory protein	AGY49269	*Sesamia inferens*	2E-60	69
CSP5	chemosensory protein 7 precursor	NP_001037068	*Bombyx mori*	1E-46	62
CSP6	chemosensory protein 2	BAV56806	*Ostrinia furnacalis*	2E-61	69
CSP7	chemosensory protein	AOG12895	*Eogystia hippophaecolus*	2E-47	56
CSP8	putative chemosensory protein CSP3	ALJ302214	*Spodoptera litura*	1E-50	62
CSP9	chemosensory protein CSP15	ATD12158	*Cydia pomenella*	8E-47	57
**Odorant binding proteins**					
OBP1	general odorant-binding protein 72-like	XP_0131366236	*Papilio polytes*	8E-79	79
OBP2	odorant-binding protein	AOG12878	*Eogystia hippophaecolus*	3E-78	89
OBP3	odorant-binding protein OBP14	ATD12155	*Cydia pomonella*	3E-70	54
OBP4	odorant-binding protein 7	AKI87968	*Spodoptera litura*	2E-71	70
OBP5	odorant-binding protein OBP13	ATD12154	*Cydia pomonella*	5E-55	53
OBP6	odorant-binding protein	AOG12879	*Eogystia hippophaecolus*	6E-57	62
OBP7	odorant-binding protein	AGK24577	*Chilo suppressalis*	6E-28	39

*Accession number of the GenBank homologue.

‡E-value for the comparison of the PBW transcript AA sequence and the GenBank homologue.
