# Supplementary material for: Pheromone gland transcriptome of the pink bollworm moth, Pectinophora gossypiella: Comparison between a laboratory and field population
Source: PLoS One. 2019 Jul 22;14(7):e0220187. doi: 10.1371/journal.pone.0220187 (PMC6645563; doi:10.1371/journal.pone.0220187)
Supplement: S5 Table — (PDF) [file pone.0220187.s007.pdf]

Table S5. Comparison of candidate transcripts of G-protein coupled receptors, odorant binding proteins, chemosensory proteins in PBW pheromone glands.

Gene	Lab population			Field population			Log2 Fold Change	% Identity*
	AA Length	Complete ORF	RPKM	AA Length	Complete ORF	RPKM		
**Diapause hormone receptor**								
DHr	521	Y	2.68±0.34	521	Y	4.98±0.79	-0.9	100
**Ecdysis-triggering hormone receptor**								
ETHr	550	Y	0.74±0.33	550	Y	0.5±0.089	0.56	100
**Octopamine receptor**								
Octo_r1	388	Y	3.21±1.09	388	Y	3.15±0.64	0.03	100
Octo_r2	520	Y	0.3±0.06	520	Y	0.4±0.09	-0.44	100
**Sex peptide receptors**								
SPr1	429	Y	1.3±0.64	429	Y	1.94±0.81	-0.58	100
SPr2	243	N	0.17±0	302	N	0.18±0.04	-0.07	100
SPr3	388	Y	2.85±0.45	388	Y	3.26±0.50	-0.19	100
SPr4	424	Y	0.39±0	424	Y	0.23±0.08	0.77	100
Lab_SPr4 vs Field_SPr5	424	Y	0.39±0	423	Y	0.45±0.07	-0.23	83
**PBAN receptor**								
PBANr1	400	Y	2.57±0.52	400	Y	2.04±0.15	0.33	100
PBANr2	470	Y	0.76±0.28	470	Y	3.49±0.37	-2.2	100
**Chemosensory proteins**								
CSP1	107	Y	0.22	106	Y	0.34	-0.09	100
CSP2	122	Y	32.1±7.0	122	Y	34.3±7.1	-0.50	100
CSP3	127	Y	2248.7±733.8	127	Y	3177.9±946.9	0.86	100
CSP4	131	Y	21.5±8.3	131	Y	26.4±16.7	-0.30	100
CSP5	120	Y	28.5±9.3	120	Y	15.74±8.6	0.86	100
CSP6	126	Y	909.3±652.0	126	Y	777.9±418.1	0.23	100
CSP7	121	N	0.35	115	N	0.37	-0.08	100
CSP8	126	Y	283.8±83.1	126	Y	332.7±199.7	-0.23	100
CSP9	122	Y	0.48±0.07	122	Y	0.46±0.19	0.08	100
**Odorant binding proteins**								
OBP1	141	Y	12.8±7.9	141	Y	14.0±5.5	-0.13	100
OBP2	143	Y	13.8±9.9	143	Y	19.7±10.8	-0.52	100
OBP3	213	N	59.9±7.2	191	Y	58.7±7.8	0.03	100
OBP4	143	Y	1.8±1.3	143	Y	0.4±0.2	2.08	100
OBP5	149	Y	1.6±1.4	103	Y	1.9±1.6	-0.27	100
OBP6	136	Y	4.8±4.4	136	Y	9.0±8.8	-0.90	100
OBP7	141	Y	147.8 ±98.9	141	Y	184.4 ±42.6	-0.32	100

* % identity between the Lab and Field populations.
