# Supplementary material for: Pheromone gland transcriptome of the pink bollworm moth, Pectinophora gossypiella: Comparison between a laboratory and field population
Source: PLoS One. 2019 Jul 22;14(7):e0220187. doi: 10.1371/journal.pone.0220187 (PMC6645563; doi:10.1371/journal.pone.0220187)
Supplement: S1 File — (PDF) [file pone.0220187.s008.pdf]

Transcripts involved in saturated fatty acid biosynthesis and β-oxidation.

Acetyl-CoA carboxylase (ACC)

ACC catalyzes the carboxylation of acetyl-CoA to form malonyl-CoA, which is one of the first reactions in the biosynthetic pathway. In the PBW pheromone gland, we found one transcripts encoding ACC with a complete ORF (amino acid length 2394), in both the Lab and Field populations. The ACC shows 89% identity to *Papilio xuthus* ACC (GenBank: XP_013176189) (Table 3). Based on RPKM value, the $\log_2$ fold change is 0.4, which shows no difference.

Fatty acid synthase (FAS)

FAS catalyzes the synthesis of saturated fatty acid (stearic acid) from acetyl-CoA and malonyl-CoA. We found 4 putative FAS-like partial transcripts. They all showed 100% identity between the two populations. FAS2 and FAS4 showed high similarity (78%) to *Helicoverpa* species FAS (GenBank: AKD01761, XP_021186732). FAS4 had the highest RPKM value than the other three FAS, and it is lower expressed in Field population (Log_2 FC = 1.14).

β-oxidation enzymes

Once the double bonds are introduced into the fatty acyl chain, the chain will be shortened by β-oxidation enzymes to form the proper length of fatty acid precursors. This step is the action of a series enzymes, working sequentially and forming a reaction spiral. The first step is converting the acyl-CoA into E2-enoyl-CoA by acyl-CoA oxidases (ACO) and acyl-CoA dehydrogenases (ACD). In PBW transcripts, we found 6 candidate ACOs in both populations (Table 3). All of them show 100% identity based on amino acid comparison and the abundant levels were similar except ACO4 ($\log_2$ FC= -1.46) and ACO5 ($\log_2$ FC= -1.17) (S3 Table). Three of them are full sequences (ACO2, ACO3, ACO6) and three are partial sequences with different length in the two populations. The amino acid of Lab_ACO5 is 87% identical to the predicted ACO of *A. transitella* (Protein ID: XP_013188649), while Field_ACO5 shows 78% similarity to the putative ACO of *P. xuthus* (Protein ID:

KPJ00249). Four *ACD*s were found from both populations. The amino acid alignment shows 100% identity and their RPKM values had no significant differences between the two populations. *ACD1* and *ACD3* show 75% and 92% amino acid identity to the short-chain ACD from *S. litura* and *H. virescens*, respectively, and *ACD4* is 80% amino acid identity to the *S. litura* very long chain ACD.

The next step is that enoyl-CoA hydratase hydrated E2-enoyl-CoA to L-3-hydroxyacyl-CoA. There are two categories of enoyl-CoA hydratases identified in mitochondria. One is specific to crotonyl-CoA (4C), the other is hydrating medium-chain and long-chain substrates. In this study, we found two ECHs with the full length of 278 and 332, homologous to the ECH of *S. litura* and *P. Xuthus* with 81% and 74% identity, respectively (Table 3). Between these two populations, the amino acids were 100% identical and the abundance level were similar as well (S3 Table).

The third step of β-oxidation is the production of 3-ketoacyl-CoA from L-3-hydroxyacyl-CoA by L-3-hydroxyacyl-CoA dehydrogenase. There are three kinds of L-3-hydroxyacyl-CoA identified in mitochondria that are specific to fatty acyl chain-length. Four 3-HCDs were found from the transcripts of PBW with full sequences (Table 3). The amino acids show 100% identity in Lab and Field populations. 3-HCD3 has the highest abundant level (RPKM ~ 30) among the 4 candidates. It is homologous to the predicted 3-HCD of *A. transitella* (Protein ID: XP_013190290) with 90%, respectively. The expression levels based on the RPKM values are very low (around 10) (S3 Table).

The last enzyme in the limited chain shortening sequence is a thiolase that cleaves the 3-ketoacyl-CoA between its α- and β-carbon atoms, making the chain two carbons' shorter. We found one full sequence of 3-ketoacyl-CoA thiolase with 400 amino acids in the two populations, and it is homologous to *B. mori* 3-ketoacyl-CoA thiolase with 86% identity. The transcript abundance level is similar between populations (S3 Table).

Identification of putative genes related to receptors

The production of sex pheromone is regulated by PBAN, which is released from the subesophageal ganglion into the hemolymph and binds to the PBAN receptors in the membrane of pheromone producing cells.

We found two transcripts, PBANr1 and PBANr2 (S4 Table), encoding proteins highly homologous to PBAN receptors from other moths. Amino acids sequence comparison shows they are 100% identical between the two populations but with low abundance (RPKM < 3) (S5 Table). .PBANr2 shows higher expression in Field population (log_2 FC = -2.2). In addition, we also found some G-protein-coupled receptors that show high homology to diapause hormone receptor (DHr) of *H. armigera* (Protein ID: ANB78221), ecdysis-triggering hormone receptor (ETHr) of *D. plexippus* (Protein ID: OWR50706), octopamine receptor (Octo_r) of *P. xuthus* (Protein ID: XP_013165403, XP_022827336) and sex peptide receptors (SPr) of *P. xuthus A. transitella S. litura* (Protein ID: XP_013178706, XP_013187671, XP_013195850, XP_022817535) (S4 Table). These transcripts all have low abundance level in both populations. The amino acid sequence comparison between these two populations shows 100% identity except for the Field_SPr5 which is 83% identity to *Lab_SPr4* (S5 Table).

Pheromone biosynthesis is initiated by release of PBAN from the subesophageal ganglion that will then bind to its GPCR receptor on pheromone gland cells. In moths 3 PBAN variants have been identified that vary in the C-terminal length and sequence (Lee et al., 2012). We found two PBAN receptors which correspond to the B and C variants. There was no difference between the populations in these PBAN receptors except for a statistical difference between Lab and Field transcripts abundance. In addition, we found transcripts encoding for four other GPCRs: DHr, ETHr, sex peptide receptor, and octopamine receptor. It is interesting to note that the DHr and ETHr belong to the same family of receptors as does PBAN (Jurenka, 2015). The sex peptide has been implicated in termination of pheromone production (Hanin et al., 2012) while octopamine could modulate pheromone production (Rafaeli and Gileadi, 1995).

Identification of putative carrier proteins

The odorant binding proteins (OBP) and the chemosensory proteins (CSP) are involved in olfaction and contact chemosensation (Tegoni et al. 2004). OBPs or CSPs capture chemical volatiles and transport them through the aqueous lymph to the olfactory receptors (Wojtasek and Leal, 1999). In PBW PG transcripts, we

found 9 CSPs and 7 OBPs (S4 Table). All of them are 100% identity between the two populations. And their expression level shows the same level except OBP4 with $\log_2$ FC = 2.08 (S5 Table).

References

1. Lee JM, Hull JJ, Kawai T, Goto C, Kurihara M, Tanokura M, et al. Re-Evaluation of the PBAN receptor molecule: characterization of PBANR variants expressed in the pheromone glands of moths. Front Endocrinol (Lausanne). 2012; 3: 6.
2. Jurenka R. The PRXamide neuropeptide signalling system. Adv Insect Physiol. 2015; 49: 123-70.
3. Hanin O, Azrielli A, Applebaum SW, Rafaeli A. Functional impact of silencing the *Helicoverpa armigera* sex-peptide receptor on female reproductive behaviour. Insect Mol Biol. 2012; 21: 161-7.
4. Rafaeli A, Gileadi C. Modulation of the PBAN-stimulated of pheromonotropic activity in *Helicoverpa armigera*. Insect Biochem Mol Biol. 1995; 25: 827-34.
5. Tegoni M, Campanacci V, Cambillau C. Structural aspects of sexual attraction and chemical communication in insects. Trends Biochem Sci. 2004; 29: 257-64.
6. Wojtasek H, Leal WS. Conformational change in the pheromone-binding protein from *Bombyx mori* induced by pH and by interaction with membranes. J Biol Chem 1999; 274: 30950-6.
