# Supplementary material for: Pheromone gland transcriptome of the pink bollworm moth, Pectinophora gossypiella: Comparison between a laboratory and field population
Source: PLoS One. 2019 Jul 22;14(7):e0220187. doi: 10.1371/journal.pone.0220187 (PMC6645563; doi:10.1371/journal.pone.0220187)
Supplement: S2 File — (PDF) [file pone.0220187.s009.pdf]

>ACC
MLTIALIVGVVLICYLFKFFVKSSEEAISSSNKMSEQEQARCSSSESDNFEKIDSEEVRQDENGEPIKFV
VDNEPESPDAATEEDGPAHEGRDSFPNAPPPNIRRRLTPAQQLALAEKRAKLRPSMSQGTVVHSHRFQEK
DFTVATPEEFVRRFQGTKPINKVLIANNGIGAVKCMRSIRRWSYEMFKNERAVRFVVMVTPEDLKANAEY
IKMADHYVPVPGGSNNNNYANVELIVDIAIRTQVQAVWAGWGHASENPKLPELLHRAGVVFIGPPEKAMW
ALGDKIASSIVAQTADIPTLPWSGSELKAEYNSKKIKISSELFAKGCVTTPEQGLQAAQKIGFPVMIKAS
EGGGGKGIRKVENPDDFNNMFRQVQAEVPGSPIFVMKLAKSARHLEVQLLADQYGNAISLFGRDCSIQRR
HQKIIEEAPAAVAKPDVFIEMEKAAVRLAKMVGYVSAGTVEYLYDPSSGSYYFLELNPRLQVEHPCTEMV
ADVNLPAAQLQIAMGLPLYQIKDIRLLYGESPWGMTQIDFDEPKQRPSPWGHVIAARITSENPDEGFKPS
SGTVQELNFRSSKNVWGYFSVAASGGLHEFADSQFGHCFSWGETREQARENLVIALKELSIRGDFRTTVE
YLITLLETSSFQDNNIDTAWLDALIAERVQSEKPDIMLGVICGSILIADSIITAHLQEFKSALEKGQIQG
SSQLSNNVDVELIHEGTKYRVQATKSGPTSYFLAMNGSFKELEVHKLTDGGTLLSVDGASFTTYLKDEVD
KYRIVIGNQTVVFEKEKDPSKLRAPSAGKLINCLVEDGGHIAKGQPTAEIEVMKMVMTLSAPESGKVTWN
LRPGAVLEMGSLMGTLELDDPSLVTTAQPYKGQFPLDDGPTVSDKLNHAHNKFRSILENTLAGFCLPEPY
NTPRLREVVEKFMQSLRDPSLPLLELQEVLSSTSGRIPISVEKKVRKLMALYERNITSVLAQFPSQQIAS
VIDHHAASLQKRADRDVFFMSTQALVVLVQRYRNGIRGRMKAAVHDLLKQYYQVESHFQLGSYDKCVMAL
RDRYKDDMQAVANIIFSHNQVAKKNLLVTLLIDHLWSNEPGLTDELAATLNELTSLHRAEHSRVALRARQ
VLIAAHQPAYELRHNQMESIFLSAVDMYGHDFHPENLQKLILSETSIFDILHDFFYHTNAAVCNAALEVY
VRRAYTSYDITCLQHLALSGELGVVHFQFVLPTGHPNRIPLSQSEIELASAQDQEGCPAELSSAAQRKCH
HRTGALASFESFDQFTQYADELLDLVHDFASSATVRREDLQALQEGSESRDSTSINVGDYKANIDADEVP
LEPIHILMIGVRDTGETDDSAVARRFGNFCRAHRHELHQKRIRRITFMSLIKRQFPKFFTYRARNDFTED
TIYRHLEPASAFQLELYRMRSYELEALPTSNQKMHLYLGKAKVKKGQEVTDYRFFIRSIIRHQDLITKEA
SFEYLQNEGERVLLEAMDELEVAFSHPLAKRTDCNHIFLNFGPTVIMDPAKIEESVLGMVMRYGPRLWKL
RVLQAEIRFTLRIGPGAPTKNVRLCLSNGSGYSLDVYTYEEVSDPRTGVIMFQSYGPKQGPMHGLPISTP
YVTKDYLQQKRFLATSQGTTYVYDIPDMFRQIVEKRWRECIEDGSVDGPPPDTVMSSVELVVEPDGERRV
VEVTRLPGQNTVGMVAWRMTLFTPEVPRGRDMILIANDLTHYMGSFGPQEDWVYYRASQYARELKIPRIY
VSVNSGARIGVAEEVKSEFNVAWVDAERPERGFKYLYLTPESYSKLGPLGSVKTQLIEDEGESRYKITDI
IGKEDGLGVECLRDAGLIAGETAQAYEDIVTISVVTCRAIGIGSYVVRLGHRVVQVESSYIILTGHAALN
KVLGRAVYASNNQLGGVQIMHNNGVSSAVAHTDLDAIKTVVTWLSYVPKDKLSTVPIMRTVDPIDRPVEW
VPTRAPHDPRFMLAGDGTKAGFFDVGSFDEVMRPWAQTVITGRARLGGIPVGVVAVETRTVELTLPADPA
NLDSEAKTVQQAGQVWFPDSAYKTSQAINDFSRENLPIIIFANWRGFSAGQKDMYEQILKFGAEIVRALR
GATAPVLVYIPPGAELRGGAWAVVDPSVNATRMEMYADNEARGGILEPEGTVEVKFKPRDILKTMHRMDP
ELLRLGSRISELKEQIKEISKNLDRRGSVDDAIIKTDAGKQAESKVRELETELLAAEKTVKAREKELSPI
YHEIAVQFAELHDTAERMLEKGCIFDIIPWRESRRLLHWRLKRLLRQNEQERRIQAAGVLPAARMDHGAA
AATLRRWFAEDRGQPQSHQWEHDNEAVCRWLEAQAGDPASVLNTNLRAIREDNVLHTVNSLVMELTPAQR
SEFIRKLSALELNQ
>FAS1
MNNISSSDLRQATLNCTAALGVALDIASSTRQDNFEFGMNNLTNDRFYYTVDFSTIFNNMNVEEIKTLQL
LVAGGIRQGFVRPLSRVTYSPQDMPRAFRLLAASRHRGRVLVDLRHSVPITHSRPEYSSDYSHLIFCDKS
DLGVQLADRLVSNGVKKIYIHSFHTSTYLQFKIIGWQEIGVQVYVSYGNLKENDSIDKILNECNGLAPIE
GIYIINIDATNARPADDLKNLVSHLDVATRQLCPSLRNFAIINENYLDREVCLNRVQDGFPVVSMELHNF
EKFSDVSLNNRGDIDHNILRLPSALDGIEKALSSKKSIILTYAKSMEKNFLVDVERLLGVNLTDTNNDNT
SLEQLGLDGGKAQELSNIIEEKYNVTISKEDYLLMTLAELKTLQEQLLNRKQDLKGLSSYINFVDDDELI
SSNEIVPKSTLVHTSDLCAVAFENELTYLCIVPGIEGHHRRFDTLCERLKLPALVLQPGIDMLDETIEEM

AQRVVDGMLKRLILKDTFYLLGYEIGVLVALEMAMILEKRGLTGVVYCLGGGPEDICATMEAQFGNIALE
ELQNQLILHMYSTIVQSKSDELQTRIKELGDWEAKVDECILLIRNRSSRSIDYIRAVINSTYRRVLIARD
YVWKQPKLSSKIVVIKAKSETESSSDSSLENVHQLDSTLPLVPEDLRCSAIVNKYLDAKILEDYYSQNLC
ETSKIETLDKVQFAETE
>FAS2
MPSAVRPTEAEEVVLTGLSGRLPESGSIEEFAEQLFAGVDMVTGDGRRWQPGLHGLPERNGKLKELARFD
ATFFGVHGKQAHLMDPQLRLLLELTHEAIVDAGVSPAELRGSRTGVYVGVSNSETEEMWTTDPDKINGYA
LTGCCRAMFPNRISYTFDLKGPSYAVDTACSSSMFALSQAVAAMRAGHCDAAIVAGTNLCLKPANSLNFH
RLSMLSPEGRCAAFDAAGRGYVRSEAAVVVLLQRRSDARRLYATVRGARMNTDGNKEQGITFPNGEMQRR
LAAETFAEAGLRPQDVAYVEAHGTGTKVGDPQEVNAIAELFCKDRKKPLLLGSVKSNMGHSEPASGLCSI
AKVVVAMEHGEIPANLHYSSPNPDIPALSDGRIQVVAQNTKWDGGLVAVNSFGFGGANAHVILESQRGGR
PAPARYAVPRLVTASGRTDDAVRELLALAQRHATDAELHALLDAVHAHNIAGHGRRGYRVLADPPLEEII
EHGGEPRPVWFVFAGMGSQWAGMARQLLALPVFAESIARSAAALRPHGLDLVHVLTEAPDSAFDDVINSF
VSIAAVQVALVDVLRELGVRPDGIVGHSVGEVGCAYADETLTGEQAVLCAYWRGRSIVDAKLAPGAMAAV
GLSWEQCGERLPADVVAACHNAADSVTVSGPVASIEKLVSELAAEGVFARRVNSSGVAFHSKYIAAAAPL
LRRSLERVIPEPRPRSARWVSSSLPREQWQSELARLSGAAYHVNNLLSPVRFAEALAQVPERALLVEVAP
HALLQAVLRRARPQAALVPLVRRDAPDALRHLLGAVGKLYAHGLQPALRRLYPAVAWPVSRGTPGLASRV
LWDHSAEWSVADFSNASRSGENVIEYDLSKQEDSFIAGHNIDGRILFPATGYLTLVWRTMAKLHNRRPEE
TAVVLEDVQFRRATIVSRDAPTRFLVSVLDASGQFEVCEGGSVAVTGTVRLAEQPAAERLALPRAAVSAD
DAELQPLLTDDIYKELRLRGYNYGGIFRGIRTSDARGTAGELAWDDNWISFMDTMLQFGIIGVDTRELYL
PTRLQRALIDPAAHAAALAARPAGQALPVRMLRDIDVVVAGGVEFRGIKTSLAPRRANPQPAPKLEKYVF
VPYDNAAVGTDDTSRSKRDALLVSLQLVLENAGALKLKAAEAALERAAEALVLPAALPLLAAEPQVRVDA
TLAAGPQPQPYQALADLDIAVTNKDPRTGGAESGCHLVLAHDVLARHGPSTLAALTGWLADTGMLLLEEP
LRQLDAPDAGDMLRKAGLVLVSRQVAASCEYVLLRRKPEPPSAHVVVEVADDGKYAWVEPLRAALARAEL
EPLRVFAVARGAAPGALG
>FAS3
TSFLHCFTSSFNPDTGGNEDRDNLEQLPELDLAFNVLVNGVWGGEYYTSWQDKVTADKAVALRSSRVGDL
ESLHWVEVGAPTEPGIPVTVHYTAINDYDVKKATGVAQIRFEKETSYCMDFSGVTDKGDRVMGVIPTGAA
CSRVKARSNLLWPVPDHWSLEEAATVPSAYAQAFYCLGVKVQFRRGYKILVHGATGALGQATINVALAHG
CEVFATVSDIRKKRFLMKLFPQIKEDHIGNSRDASFGDMVRNLTKDKGCNIVINCLRGQLRNVSMNCLSM
CGILVDTIQIPHQEDYNFGLFYLTKERNYTPIDFASVLTENSKDLQKIQILVSEGIARGYVKPLTRMTYA
PEHVSRAFRLFAASRHRGRVLMRLQGKGTVAEPRLTCSPELCHVVICDENIFALQWTERLISRGARKVHL
HCGHPSKNLQLKVQTWQSQGVKVQVSSEVLMNNKGIVSVLSESNRLGPVEGIYVIATDASEKSVDKISAV
VANLDLAARKLSPSIRFFAVVNAGISVGQQTCGVRVRDGLPATVLNLPALRKPHDVNTTKETISWRSAVN
ATERAIRSSQSNLLAHPLTVPRPSLLQQIAAISDIEISEETAGNITLLDLGIEVAKVPAICSFLQDTHNV
TLVEEKIPNLTIQNIRALEEIATGIKFEKVRGLKTFFTYVDPDELLATTEMVFLPTLTNSSTMREDEFNV
NDTFLCIIPGMEGHHHRFQVLCERLKLPALVLQPGLDHLRETPRETAERYAKNLLKKACVRSNFYLLGYE
SGVLVALELAAILEDHGLIGTVFCLGGTPEEFLALLDEELSCIPNDEALQDAVIHHMYTLMTGSEEGLDK
ALENASTWHEKVESCVRMLLGRVAHSAQYARSLLQAAYARLKQGQTYKLQPRELKSKLVLLHAANSHTDV
SPAKLLQRYSQHPVTVHELCEPLAHCTRDLRCSAIINSSIDPELIKSFNERNICDTYLMNADTFMTVVTD
ADNIE
>FAS4
RLAAPGALGLAACLRGEAGGRALRLFVAPGLQAPFSLAAGPFAAQARLDLSVNVLRAGVWGCYRHLPLAD
ASAAQLQVEHAYVNTLTRGDLSSLRWIESELRWAREAPARPGTELCRVYYAPLNFRDIMLATGKLPPDAL

PGDLAGQECILGLEFSGRGADGRRVMGMVAARGLATSVLADGGFLWPVPERWSLEEAATVPVAYATAYYA
LLVRGRMRRGESVLVHAGTGGVGQAAIAIALHAGCTVFTTVGTPDKRAFLRERFPQLDEANVGNSRDTSF
EQLVLTRTRGRGVDLVLNSLAADKLLASVRCLAPGGRFLEIGKLDLSNNTPLGMAIFLKNTTFHGILLDA
LFDAGGDNEEKAAVVRCMNDGIASGAVRPLPATVYADHQLEPAFRFMATGKHIGKVVLRVREEEPSGPAA
APRLVSAMPRTYMHPARSYVLVGGLGGFGLELAQWLVVRGARQLVLVGRSGVRSGYQAWCVRRWRARGVR
VLVSRTDAATPAGARALLRDAAALAPVGGVFNLAAVLRDAFLENLAPDDFRAVAAPKLDATRALDEATRE
LAPQLEYFVVFSSVSCGRGNPGQSNYGYANSCMERICEQRQADGLPGLAVQWGAIGEVGLIVDTMGGDDT
EVGGTVPQRIASCMEALGTLLALPHPVVASMVLADKRRQAKSGDSGDLLHAVANILGVKDPSAVSQTASL
AELGMDSLMGAEIKQTLERGHDLVLGVQEIRALTFGRLRELAGGDNADAAADPAPANGAAAAGADDNVQY
GTLGELVPKQVIVKLPSAGGGRADAKPVFMVHPIEGVADVLRGVAAGVRGAVYGLQCTAAAPLADMAALA
RFYLEHVRALQPEPPYTLLGYSFGASVAVEMALQLEAAGCAARVVLVDGSPAYVATHTTRGKAKRAKRSP
AADEADALAYFAQLFKDVDAVKMSAELERLPGWEQRVSRCVELVSSAAPHEAGALAAAAESFYRKLVAGD
TYRPASKLQAPVSLFTARDNYVTLGEDYGLREVCAGPLSTQQLAGTHRSILAGESARTIAEHVSALLEQH
>DES1
MDIALVDSQAESLLVAYTGSEGVTVGAHRLYSHKSFKAKPVLRAILILVQTIAGQNCIFIWSRDHRLHHR
YSDTDADPHNSKRGFFFSHMGWLMTKKHPYVIEIGRKIDMSDLAADWMIMFQKRYYYYLYVPLSIILPVW
VPVRYFGESMRTSFLVAYIFRWMTSLHGTWLVNSAAHLYGTRPYDKNLQPVESWFVSWLSLGEGWHNYHH
VFPWDYKAAEFTRHYNFTASLIRFFERIGLAYDLKTASPEMVDKRILRTGDDTHEHWGTEAARAAVAVDW
GPLHPFNPSYNVTYPPPNSTVVEAGLPLYHEDDILLDKKER
>DES2
MPPQGQPSFVLFESDAVAEDKVPDVAPSAEAEKRKLRIVWRNVIIFAALHLGGVYGAFLFLAKAKWATSI
FAVFLYICSGLGITAGAHRLWAHKSYKARWPLRLLLTVFNTMAFQDAAIDWARDHRMHHKYSETDADPHN
ATRGFFFSHVGWLLVRKHPEIKNKGHTIDMGDLWADPILRFQKKHYLVLMPLFCFVLPTIIPTLWGESLW
NGYFVCAIFRYVYVLNVTWLVNSAAHMFGTKPYDKNINPVENKSVALVVFGEGFHNYHHTFPWDYKTAEL
GGYSLNLTKMFIDTMAKLGLAYDLKTVSHEVIEKRVKRTGDGSHDVWGWDDKDVPAEHKEITTIVNPEKT
E
>DES3
MAPNTKTSESLLKTPEDLKSSEDTKQKLVHLNLLTFGYGHLAGLYGLYLCFTSAKWPTIFLNYVLLIISE
LGVTAGAHRLWAHKTYKAKLPLQILLMIFNSVAFQNTATDWVRDHRMHHKYSDTDADPHNSNRGFFYSHV
GWLLMRKHPEVKRLGKGIEMRDIYSNPVLRFQKRYALPVIGAACFILPTVIPVYCWGETLSNSWHVTIFR
YVVNLNVTFLVNSAAHIWGNKPYDKTLRPAQNIPVALLTLGEGFHNYHHVFPWDYRTAELGNNKLNVTTF
FINFFAWIGWAYDLKTAPDDIIRKRVERTGDGTNMWGWGDKDMSKDDRESVKILGEKFL
>DES4
MAPNLKKGPTIYENGQAEEQLEKLVPPQANPWKYQFIYPAVLYLITTHVLGVYALYCLYYNWKSVKLATV
LYNMAVYVFAMQGVTAGAHRLWSHRSYKATTPLKVLLVLGQSMSMQYSVLWWAITHRLHHKFSDTDADPH
NSARGFFFCHIGWLMVKPHPEALKRRKFVDMSDLKRDPLLVFQRKHMLIITIIMAYVVPILTPVIFWSEN
FFLSWHINKLRIIMVMNATFSINSFAHRWGTKPFDKSIEATSSGLVSNLTLGEGWHNFHHVFPWDFSHDE
LGSHLDLTTKVIKMFAKIGWAYDLKTVSRDVINIRTSRCGDGTIIKKAE
>DES5
MEKSKVDPRPSLSEIFRTFEKSLGFKNDIKWDNFILITLYHIIGMYWSYYYAFPVKWQTLVFAAIMYVAS
GFGVTGGAHRLWTHKAYKVKLPLKLFLLMCFSSAGQNSLLQWVRDHRVHHKFSDTEADPHNANRGLFFSH
IGWLMMKKNEEVLRRGKQIDMSDIENDPILKVYNRYFGFFKLFFCYVLPTMIGIWLWGEEWKCAVAWQCF
IRYLTVFHSELTVNSLAHTFGYKPYNKTIMPAENRFVSTCSLGEGWHNYHHAFPFDYKAAEHFDIFNFGA
RFIRFFGKMGWAYDLREATPEMINGIAERMGDGTPVHFPLKSDSHENRACG

>DES6

MSPNSKNNGDILPERQLERLVAPQASPRKFQIVYFNILTVGYTHLAALYGIYLCFTVALWKTLLLNFVVG
VFAAIGVGAGAHRLWSHRSYKAKFPLEILLMIFHSMAFQNTVIDFVRDHRLHHKYSDTDADPHNATRGFF
YSHYGWLLVKKHPEVTRRGQYTDMSDIYSNPVLNFQRKYAIPLIGTVCFILPTFLPVYFWGETVKTAWHI
CMLRYALNLNFTFSINSFTHMYGYQPFDKNILPVNNFLANIVTLGEGHHNFHHVFPWDYRGTHVSNLWNI
SCLLIDLFAWIGWAYGLKTVPEEIIQARIKRTAKMDSI

>DES7

MGQKVSRTDFEWVYTEEPHASRRKIILEKYPQIKKLFGHDPLFKWVVTLMVVTQLCMLPVVQQLSWPATL
VLAYCFGGVINHSLMLAIHEIAHNLAFGHNRPMANRLFGFFANLPIGIPISISFKKYHLEHHRYQGDEVI
DTDLPTLLEAKLFNTTGGKLVWLFLQPLFYALRPLVVRPKPPSPLELINLIIQLFFDAVVVKLFGWKVLG
YLIYGSLMAMGVHPVAGHFISEHYMFRKGFETYSYYGPLNWITFNVGYHNEHHDFPAVPGRRLPEVKRIA
PEFYDDLPQHNSWSSVLYDFVTDPDIGPYARIKRKHHGLDS

>DES8

MANPDSEAVPFRHTLVKHNVIKFGYMHLAALYGVYLCFTSAKWPTLLWAFLLLECAKIGITAGAHRLWCH
RSYKAKLPLEILLLIFNSIAYMNTATYWVRDHRVHHKFADTDADPHNVNRGFWFSQIGWLFVRKHPDVLN
KGQTVYMEDIHKNPLLRFQKKYAFFVIGLWAYVIPTVVPMYFWGESFNNSWHICTMLRHVLTINQIFLVN
SIGHSWGNKPYDKNIRAVENIAVSLMSTGECFHNYHHVFPFDYKASELGMTKFNAATMFINFFAWLGWAY
DLKTIPDELIIARSKRTGDGRNLWGWGDTDQTEEETNGVKYLYQKGE

>DES9

MVQISEATEGPQEGQRVSRSREANWPAVLFFIHIHLLSAYGLWLLFFEVKLMTILLLITLTLFAVLGVTT
GAHRLWAHRAYKATPALRIFLAFCQTLAGQGPIYDWVRHHRLHHAQFGTNGDPYDHRQGFLYAHILTRMR
KLSPHQDKLLEQIDMSDLESDSIVMWQKRLYWILYVVFFLLLPLNAPLEYWDDTVLASLFVVGFLRYGII
LHAAWLVESGICVWGLQPGEKYPHDSNSVFILAKTFWPHYHYLIPYDYKSGEFGTYDCGCSTAFIRVWAA
LGLATDLRTVETPAIQQALADAAKNKRPVKDSIDDAAAKQTLPAEHYLKRG

>DES10

MPPQGQPPLVYFDAETTKTKAPVVHTPEQKREWKIVWRNVIVFTLLHIGSVYGAYLFLFQAKWATSLFAV
TLYICSGLGITAGAHRLWAHKSYKAHWPLRLALTVFNTIAFQDAAIDWARDHRMHHKYSETDADPHNATR
GFFFSHVGWLLVKKHPEIKAKGHTIDMSDLWADPILRFQKKYYMILMPLACFILPAVIPTLWGESLWNGF
FVCSIFRYVYVLNVTWLVNSAAHMFGNKPYDHNISPVENKSVALVVLGEGFHNYHHTFPWDYKTAELGGY
SLNITKMFIDAMAKIGLAYDLKTVSHDIIEKRVKRTGDGSHDVWGWDDKDVPIEDKEITMIMNPQKLSKV
F

>DES11

MSPQMEASWALYESDIAGDKIPVVPASAKMREWHIVWKNVIIYTLLHIAAVYGGYLFFFEAKWATCAFAY
MLYIMSGLGITAGVHRLWSHKAYKANLPLRVILALFNTIAFQDAVLHWARDHRMHHKYSETDADPHNATR
GFFFSHVGWLMVKKHPEIKAKAHTIDISDLLADPVLTFQKKYYFILMPLCCFILPTIIPTLWGESIWNGF
FVAAIFRFIYVLNVTWLVNSAAHMFGSKPYDKNIKPVENKTLSVLVLGEGFHNFHHTFPWDYKSSELGDY
SLNFTNLFLDTITSTRYAND

>DES12

FNTIAFQDSAIDWARDHRMHHKYSETDADPHNATRGFFFSHIGWLLVRKHPELKKKGQGLDLSDLYADPI
LRFQKKYYIYLMPLACFVMPTVVPVYLWGESWTNAFFVCALFRYAFILNVTWLVNSAAHKWGDKPYDKSI
KPAQNLSVSVFALGEGFHNYHHTFPWDYKTAELGNNSLNFTTNFINFFSKIGWAYDLKTVSDEIVKARVE
RTGDGSHHLWGWGDKDHDQKEINAAIRINPKDD

>DES13

MPGSLPLLANAFVTKNIDDDMPEVDTNANYGKRRDYEWQIVWRNVLAFVYLHGAAVYGFYLIFTGKVMGL

TLLFALLFSALSGMGVTAGAHRLWAHRAYKAHWSLRLVLALLQTMAFQNHIYEWVRDHRVHHKFTETDAD
PHNAKRGFFFSHMGWLMVRKHKAVFEKGANVDMSDLEKDPIVMFQKKTYMVAVPLFCFILPAAVPVYLWG
ESPWISWYAAAITRYTLTLHFTWLVNSAAHIWGNKPYDRYIGATDNKAVAICAFGEGWHNYHHVFPWDYK
AAELGDYSTNLSTLLIDVAAKMGLAYDLKTVSADMIRKRVGRTGDGSHPLDKEVEHTVDHHEHPENPVWG
WDDRDMPDEERQLAEIVHRKDD
>DES14
MAPAQQNVEVCDESLHPEIKMRIAYRNKNEVEHCENNNSVLPASEKVNSETEFNIEEYEAMDFVAQIRWP
DLMAQVSLHLVSLYGLYLIFTCQVMVYTLIFVICTIYTSGFGITAGVHRLWSHRAYRAKTPLRILLAFLF
TITGQRDIYTWALDHRVHHKYAETVADPHDVRRGFWFAHVGWLVLTPHPAVENRRVALRAASPDLMADPV
VRIQSLTYIPLFVILNVILPVWIPMHFWGESLVNSFVISFVLRFTITLNIAFCVNSVAHLWGNKPYDRFI
KSVESKMVSLAALGEGWHNYHHVFPWDYRTSELGRVNLSTGFIDLFAKLGWAYDLKAASPSMILSRAKRT
GDGTLGEMEEPDPTIKDFNE
>DES15
MAPNVRVEDNIESLEDDKFVKLVAPQASPWKFELLHPVVLYVFSIHVASVYGLYVSWSSVKWATVAFTLA
YYVTSVQGITAGAHRLWSHRSYKAKLPLQILYMIAQSATMQYSIIWWASKHRQHHKFSDTDGDPHNATRH
IFFSHIGWIMTRPHPEAVKRRKLVDVSDLKSNSVLAFQRRHMVLLNLLLAFLLPIIIPVYLWSENIYTAW
HFNIFRVTITYHCIFFVNSIAHKWGMRPYDKKMQATQAPFVAHFSFGEGWHNFHHVFPWDYSSDELGSKF
SFTTKVIDFFAKIGWAYDLKSVPKEVIYDRAHRTGDGSPVMKSVS
>Gilat_DES16
MANAMEDKIKLNIIWTSVIFFSLLHIMAVYALYLVFFEGLALTTIWVYVYGLLGGLGVTAGAHRLWTHRS
YKAKLPLRVMLMLFHCIAIQNTVLDWARNHRMHHKYSETDADPHNATRGFFFSHMGWLMVRKHPDIKAKG
HTIDISDLTSDPVLQFQKKYYGVLVLLMSVVVPMYIATLWGETLWNSFFIDVIYRFTISLHHTWLVNSAA
HMWGNKPYDKHINPAENKLVAICAFGEGFHNYHHAYPWDYKTSELGSSLNFTKVFIDFMAGIGWAYDLKT
VSSEIIEQRIDRTG
>Shemer_DES16
MANAMEDKRKLNIIWTSVIFFSLLHIMAVYALYLVFFEGLALTTMWVYVYGLLGGLGVTAGAHRLWTHRS
YKAKLPLRVMLMLFHCIAIQNTVLDWARNHRMHHKYSETDADPHNAKRGFFFSHIGWLMVRKHPDIKAKG
HTIDISDLTSDPVLQFQKKYYGVLVLLMSVVVPMYIATLWGETLWNSFFIDVIYRFTISLHHTWLVNSAA
HMWGNKPYDKHINPAENKLVAICAFGEGFHNYHHAYPWDYKTSELGNYSLNVSTLFIDVMAKIGWAYDLK
TVPHEIIKQRIERSGDGSHVKFN
>DES17
MPPATITEVSLVQKPSHSDNEDNGLKLTHRLIEAKTRDTIQPSYKPNVYEETRPKNDPKFLAPIREWEKR
MGFETALRWINLIIISLFHVISVGAFLYYLLTGNFPKLQTVLFSMFMGEVAGFGVTAGAHRYWCHRSYKA
KMPLQIILMLCYSLAGQNNIYNWVRDHRVHHKFSETSADPHDARRGLFFSHVGWLMMKKHPHVLREGAKI
DMSDIVNDPLVRFHTKYFNWFKLVVCFILPTLVPVYAWGETWSFALLSQPFLRYMFSLNFTWSVNSFAHI
FGHKPYDRFIKPAENWFVSLVAMGEGWHNYHHTFPWDYKAAELGYTLNLTTLLLDGFARIGWAYDMKEAS
TTLVRTVAMNRGQRQPQ
>ACO1
QSSNLPFTYGLVTAACTYEGDNTVLLLQTARFLVKTWQQIDSITLTPTVAYLKTAATDCSPWRNTVEGII
RGFEIVAMGKISSCVKSMKERATAGASLEDAWNLTSVQLVAAAEAHCRVFILTTYYEEAYRRAAQASPEL
RVMIHQLVEVYAVYWALEKLSDLLRFTSITSQDVENLQSWYEELLLKIRPNAVGLVDAFDIPDKILQSTL
GAYDGRVYERLMEEALKSPLNQQPVNDSFHKYLKPFMRGKL
>ACO2
MEQKMGYVTDDQLKECIPDLPSGPLEKYRKQATFDWRRMKLVYDSLSTIQLKKHVWDFMRTHPLFQHQSA

SQTLDEERHIAVKRMYAIYNETFVPDLKLVDDPGLFQAQSEAMFMFDSALAVKVSLTFKMFANTIQGAGR
DHHFNYLDDIEKARIGGCFALTEISHGSNARGMRTTATYIPEKRQFILHTPDFEAAKCWVGSLGKCATHA
IIYAMLMSKGKNHGLHTFVVPIRDPETLLPYPGVTLGDLGEKIGLNGVDNGFVMFNNYHLPKEAALDKLG
GVDDNGDYQTPFRDPSKRFGASLGILSGGRVHITSISTAYLQKAIVIAIRYSAVRKQFGPPNSEEMPVME
YQQQQIRLLPYLAATYALRVFCNWFCRTHIQLTINRFMGVDDNAAGRGIEMHALSSAVKPVCGWTARDGI
QNCREACGGHGYLKAARIGDLRNDNDANCTYEGENSLLLQQASNWLLNVWSRRGTITKDDTPYGSLQFLD
NADQLLVETCPWNTVEELIDPANLTHMYKWLTAYMLKMTHEKVSTLKSSGKDKFSAKNDSQSFNAVTLSV
VYGENFIVNHFYKTANAFEDARCRNVLLKLVSLYGAFMLEKHMATLYIGGFFSKDQGLLLREGILRVCAA
LAPQAVSLADTLAPPDFCLNSVLGMADGEVYKHIQSSVMSYPGSMSRPEWWRDVVFWRSYIPSKL
>ACO3
MTTKVNSDLQRERDKCTFNITELTNIIDGGPEKTEDRKRMEELVLKEGIHHDDVPMQYLSHKEKYERAIK
KSCYLFKLIRRLQEEDNASMENYRAVLGGNLGSAILQDGSPLTLHYVMFIPTLMGQATVEQQAYWIGRAF
NLDIVGTYAQTELGHGTFIRGLETTATYDPKTKEFVLHSPTLTAYKWWPGGLAHTANYCIVMAQLYTKGK
CHGIHPFIVQLRDEETHMPLPGIKVGEIGAKLGMNGTNNGFLGFEHVRIPREHLLMKNSKVLEDGTYVNA
PSSKLTYGTMMFVRVVIVNDMCNYMAKAVTIATRYSAVRRQSQPKPNEPEPQILDYVTQQHKLFIGIASV
HAFRLGAEWIWNMYNNVTAELEAGDMDRLPELHALSCCLKAVSTSDAAETVERCRLACGGHGYMQSSNLP
VAYGLVTAACTYEGENTVLFLQTARYLVKAWQQAAGGDALPPTVSYIGRVTTGRRVPPWDNSIQGIIDGF
QRVAAGKIGLCVANIEKRQKTGMAYEDAWNMTSVQLASASEAHCRAMLLSCYYEETEKLCAQASPALRQV
LSQLADLYVVYWALQRVGDLLRFTSISERDIEQLQNWYEDLLVKLRPNAVGLVDAFDIKDEILHSTLGAY
DGRVYERLMEEALKSPLNKEPVNDSFHKYLKPFMSGKL
>ACO4
MGKINEDLQRERDRCTFNIEELTNFKDGSPEKTKLRKQREKMVHDIAEFYEGTPEEYLGHKEKYEDAIKK
HVPFFNLVRKLDKKGESLLAQYSDHIIAMMASTPSRDGTPLGGHWVVFMPAILSQGTEEQLDYWLQRASD
CSVIGTFAQTELGHGTFIRGLETTATYDPETKEFVLHSPTLTSYKWWPGGLGHTANYSVIMAQLYIKGKC
YGVHAFIVQVRDEETHMPLPGIKVGDIGAKMGINVMNNGFLGFDHVRIPRNYMLMKHAQVLEDGTYVKSV
HSKLIYGSMVFVRVVIVFDMANHFAKAATIATRYSAVRRQGQIKAGEPEAPIMDYLMQMHKLFVGISASY
AFRITATKLWDTFHDINNQLIGGNLERLPELHAISCCLKAISTADAASYVERCRLACGGHGYMQSSNLPF
TYGLVTAACTYEGDNTVLLLQTAR
>ACO5
LDLQIIGTYAQTELGHGTFIRGLETTATYDPETEEFVLHSPTMTSYKWWAGGLGRTVNHCIVVAKLFTKG
KCYGTHPFIVQIRDLETHMPLPGIKVGDIGPRMGFNTADNGFLGFENFRVPRENMMMKHAQVLKDGTYVK
VGRHEKLTYGTMVFVRVTLVNEAAFNLAKAVTIAVRYSAVRRQSQPKPDEP
>AC06
MEDLSFVPNLPSGPLDVYRNSASFDWKRLKLALEGDIDLLKLKYKIWRTLEKDPLFAHNTVTPSVEEQKR
ITQLQLKRINEYKFLTDDMFKAGYSKRTRALMTINEAVQSLNPSVSVKMAIGIYLFSNALLSLGTERHRK
FYEATISKREILASFALTEIAHGSDARLMRTTATYDPTHRQFVIHTPDFEAAKCWVGNLGRTCTHSLVFA
QLITPDGTNHGLHGFVVPIRDPDTLETYPGLIVGDMGEKIGVGGIDNGFIMFNQYRIPRENLLNRTADVT
EEGVYESSFSEPGRILGAALENLSAGRIGIMQESCHSIASVVAIAIRYSATRKQFGNRNDEIPLLEYPLH
QWRLFGHVAAAVVFRIYIESFTRVYLNIIEKSNAGMKVDNLSAAVSEIHAMVSCSKPTITWETLHAAQEC
RECCGGHGYLKCANLGEIRNNHEPTVTYEGDNNVLQQQAGNWLLRQWELALNGNPVDSPLGTVEFLNDYS
KILATKFHCTETSQLTPEFITATYKWLICWLLRHTHETYETELNRGLSKFQAKTKCQVYRSRTLTRAYAE
YLALIFSLKSIEKKEKSLQPVLYKMFALFGLWSLDKHLVELYQGGYCGGEACARLVREGVLELCGQLRGE
VVSVVDALAPTDFVLNSVLGKSDGKLYQNIQKAFFSQPGVFERAAWWRDVVPASKL
>ACD1MAGGVFFKSGKFALSSVQCTKRLVSRTFTTQLTEQQQSMQELARNFADKHLKPHA

GALDRAGLFPFEPIQKLTNLGLMGTSVDGEYGGLGLDYLSLALVVEEISRGCASTGMILSIHNFLYANLV
NEKGTEEQKELFLKKFTRGAIGCFALSEPGAGSDAANIKTTATADGDYWVLNGRKSWVTSSIEGSAAVVF
ATFDPELRHKGIACFLVPLDTEGVFRGNKEPIIGVSVCDRAATACDVTLSDVRIPRGYMVGQQGEGFKIA
MEQLDQGRIGIAAHAVGIAQAALDTAMNYAKQREAFGKSLTRLASVKDRLTEMCMLVETARLLTYRAATD
VSTKNSAMAKYVAGRNANAVADHCVQILGGRGLSTKYSAERHFRDARGTQVYGGVTDIQKRLVGHYLLKE
HDAL
>ACD2
MNIARKICTIRHQNLTRPFVRKLRLSASNFESSQATQPQVKEDKFDFEDISILERTERRKAKIPPFMKDV
FVSVFNRDMLAYPEVINKEETEQLEYRLSALDKVFSDKEKTKDDRVEALKKTKMYAGPVSLTKGGLAMNA
TESLRYLEVIASDTQLGQQISDHWVALEALKIGLTSDQYQQIIDDLTSGEKTIGLCIKEKVAERITQADF
RTTAEMDGQGIWRISGEKLCYYNEGYLLVLTVTETGRFKALLVHPGAQGLTKDGDFVSFIKTPGTPLELA
SDAQLAQTLGLSRLHAATLSRSNLTKTLHVCGEYVHSKVFCGKPLAELSTIRQAFGLALLDIYASESAEY
FTAGLMDAYEQPDTELEAAMCRNFMATHAMPSVLSLLAIPALEWPSVSGTLLDEARHLILRGETLHSVNM
FIALNGIHHAGKLMAQEVKQVRNPLFHPTFIIKKVIANRLQEKDAPKLTLYLAEHLHPSLRQPAEQLEYC
VLRMKYACDTLMTRHGQDITTAYTELDRLAEAATEILAMTAVLARASRAYCIGLRNAEVEMKLAACFVER
TKDKVKKLILEIDDGEYLNFDHFTLQFGKKILDTNTTLVEKPTARVYW*
>ACD3
MFGAISRSVQVFAPTAVRQSRCIASLSALPDTYQMLYKTCRDFAEGELKPNAAKYDRDHLYPGEAIKKMG
ELGLMSIAVPEELGGAGLDYLAYAIALEEVSRGCASAGVIMSVNNSLYLGPVQGWGTDKQKQEYITPFCG
GDTIGCFALSEPGNGSDAGAASTTAKDGGDKWILNGTKCWITNGYESKASVVFATTDKSLKHKGISAFIV
PKPIKGLELGKKEDKLGIRGSSTCSLIFEDCNIPKENILGQPGLGFKIAMMTLDAGRIGIASQALGIAQA
SLDVAVDYASKRIAFGKPIMKLQAIQNKLADMAMQLESARLLTWRAAWLKDNKKPYTKEAAMAKLAASEA
ATFLSHQCIQILGGMGYVSDMPAERHYRDARITEIYEGTSEIQRLVIAGQVIKEYGF
>ACD4
MRSARLWSSGMGRCAAPCRALSGSGPRAAEARPAPARAARSSTSFTLNLFRGAMEPAQVFPFPEPLSPDQ
KQMLAELVPPFEKFFQEVNDPARNDQEASIEEGTLSALWELGAFGIQVPVELGGLGLTNTQYARLVEVVG
AHDLGVGITLGAHQSIGFKGILLFGSPEQKARYLPRVTGGEFAAFCLTEPSSGSDAGSIKSRAVPAADGS
HYVLNGSKIWISNGGIAEIMTVFAQTPVQKDGKTVDKVTAFIVERSFGGVSSGPPENKMGIKCSNTTEVY
YEDVRVPAENVLGGVGNGFKVAMNILNNGRFGMAAALAGTQRAALRQAAEHAATRTQFQRKIKDFGTIQE
KLARMAMLQYTTESLAYMVSGNMDTGAQDYHLEAAISKVFASDAAWASVDEAIQILGGMGFMKATGLERV
LRDLRIFRIFEGTNDILRLFVALTGIQYAGAHLQELQRAFKNPAANLGLIFSEGAKRAAGAVGLAGGPDM
APHVAPPLAPVANELGKLVGEYGRCIEQVLRRYGRGVVDEQFVLNRLAAAAIDVYTAAVVLSRASRAARL
ALPSAAHEARLAEAWAE
>3-HCD1
MYKGMVSLVTGGASGLGKATAERFVKNGGKVVILDLQATRAQEVAKRLGNNVAVSAGCVTSEEDVKKALD
LVYDKFGRLDTLVNCAGHAATYQVYNFLKNKECVLDGFVKCVNVNTIGTFNTIRLAAGMMGKNTPDENGQ
RGVIVNTASTIAYEGDIGQAAYAASTAAVIGMTLPIARDLAPQGIRVVTIAPGIFDTPLLSYLPEKMIEF
IKRMTPFPSRLGKPEEFAHLVTSVIENPMLNGEVIRLDGAQRWFPL
>3-HCD2
MFKGLVGLVTGGASGLGRATAELLVKQGGRVIICDLPTSKGQETAKQLGENAAFVAVNVTSEEDVKKALQ
ATQDKFGRLDVAVNCAGIATAARTYNFKKEQPFDIKAFQKTVEVNLIGTFNVIRLAAGMIGKNAPDGDGQ
RGVIINTASVAAFDGQIGQAAYAASKAGVVGMTLPIARDFSKQGIRVVTIAPGLFRTPILEMLPEPKIKE
LEATVPFPSRLGHPSEFALLVKSIIQNPMLNGETIRLDGSLRMQP
>3-HCD3

MSQFRVIARNFSSSSAVQSAIKNVTIIGGGLMGSGIAQVAAQAGQNVILVDVSSDVLAKAQKSISTNLGR
VAKKVYKDNPQEGEKFVTEALGRIKTATDPAEGAKNSDLVVEAIVENLDVKHKLFKQLDAAAPAHAILAS
NTSSLSINEISSVVKRKDKFGGLHFFNPVPVMRLLEVVRGAETSEETYTSMMTWGKSVGKTCITCKDTPG
FVVNRLLVPYICEAIRLFERGDASARDIDIAMKLGAGYPMGPLELADYVGLDTNKFILDGWHKKYPNEPL
FSPIPLLNKLVAEGKLGVKTGEGFYKYEKKQK
>3-HCD4
MNKLVGITRNFSSSATQNAIKTVTVIGGGLMGSGIAQVAAQAGQNVVLIDAYPEQLEKAQKSIQGNLTRV
AKKQYKDDTGKIEGFVKESFGRIQMSTKVEDGVKSDLIVEAIVEKLDVKQELFNKLDQLAPAHTILATNT
SSISINEIGAGIKRKDRYGGLHFFNPVPVMRLLEVIKGDHISEETYQAMMAWGKSVGKTCITCKDTPGFV
VNRLLGPYSAEALRMYERGDASKEDIDIGMKLGAGYPMGPFELADYTGLDTNKFALQVMYEKTGNPVFKP
IPLLDRMVAEGKLGIKSGEGFYKYNKKKCTGIANTPCONTIGMITOCHONDRIALFULLMSVAINKGVFI
VAAKRTPFGRMGGLLKDVHPSDLVATAAKDAFKAGNVSPALVDSVNVGMVNVLSGTSDGGLSPRHAALKS
GVPQEKPALGVNRLCGSGFQAVVNSAQDIITGAAQVSLAGGTENMSAVPFVVRNIRFGVPLGANIEFEDS
LNKGSLDTYCNFTMPQTAENLAEKYDLKRGEVDEFALQSQRRWKAAFDNGVFNAEISPVTVKVKKQQVVV
DRDEHPRPETTADALAKLPVLFRKSGVVTAGNSSGVNDGAGALILASEEAVKQHGLTPLARLLGWSYVGV
DPSIMGIGPVPAIQNLLRATGLKLNDIDLIEINEAFAAQTLACVKELNLDQSKLNVNGGAIAMGHPVGAS
GARITAHLAHELRRRGLKKGIGSACIGGGQGIALLLESV
>ECH1
MLTAIQRTLRRPVLVFSRGAHTQYVQVDENNGAREITLSHEKTKNSLSLEMMEHLTEAINKNKDDTSLRA
IILSAKGNVFSAGHNLKELQASTGIDHHKLIFNKASVLMKSIVQSPVPVIAKVNGFATAAGCQLVATCDI
IVCSETSKFSTPGANFGVFCSTPGIAVGRSIPKSRAMYMLLTGEPLSAQEAYESGLVTKVVRADQLDEEV
NQIIDKIRHKSRSVIALGKQFYYKQIDLSLLEAYKLGEEIMVQNINANDGQEGIRSFVEKRKAKWSHT
>ECH2
MRVIIRRLVSATNASCTRYNTARYSSNDAKNKKKEVEEAAEKKIEEVRKNIIVEKYGGITTLNIDRQQKR
NSLDEATLREIEAAIESFDKDQQAKVLVINGEGGTFSSGFDMDEMGTKGYNVLKDAANRLRRRPLCDKPT
IAAVAGFAVAEGFELALACDLRVIEDTAVLGCLGRRFGAPQTLYGARRLTSLIGLSPALTLLMTGKLISG
VEANQMGLACKTTSTGTSLGEAIKLAKSLVKFPQNALIMDKLAAVNSTLNPNSEDSMRDEAVMESLLGNA
IEDMKEGVKKFQGGIGKHGKFYKLTEVPLKDWELEESVEEITIIEEKKDDKR
>FAR1
MPSQVNEWYRGRTLFVTGACGLMGKVLIEKLLYSVPDVGTVYALVRSKRGKTPEARIEDMWKLPLFARIR
EEKPHVMKKLVPVVGDIAYDNLGIEDKMLEKLYAEVSVVFHFAASLRLEAPLKEGLEMNTRGTLRVLHVA
SRMKNIAAFLHLSTAFCYPDYERMAERVHDPPADPHEVMRAAGWLTDKQMALLAPSIMGKHPNSYTYSKR
LAEALVREYYPRLPAAVVRPSIVTPSYKEPTPGWVDNLNGPIGLMVGAGKGVIRSMHCYGHYHAEVIPVD
LAINAIIVIAHRIGTMTERPPEIPVYNITSGDDKNTTWKDVLDIGKATVRKYPFEGPLWYPDGNIRHNKF
IHDLCVFFYHIVPAYLIDFLMFVFRQKRFMVRIQNRITVGLEVLQYFTTREWWFDTHNFKGLPATLNAED
FKTFPMDVTVIQDVPYIESCMIGGKLYCLKEKLEDLPKARLQNNILYVLDRIVTIFFYLLLVYWIVSYFE
PVRELLSYGGPVVRYLPLVGKAVFKDEYL
>FAR2
MAPQHSEGDLQTLPDRVAATFSHKTVLVTGGTGFMGKVLVEKLLRKCPDINQIILFVRPKKGKDPQERLQ
VMFSDPLFEKVTNMRGGIETLMKKMKVVGGDVQEPDLGLLPADREYIVDNVDIIIHAAATIRFDEMLKKA
VLLNVRGTKLILELAKTCKKLKLFVHISTAYCHLHEKLLEEVAYPPPADPHKVIAAAELFDEATLESMSK
KFLDTIPNSYAYTKALGEALAVEATAHIPVLILRPSIVIPVIWEPLPGWTDNINGPTGLLIGAGKGVLRS
MYCRSDSYADYLPVDVFISGIMVAIWNYLSNGDTSSSIYNFTSSAEVKVTWHEMIETGRAIILNKVPLNG
VAWYPGGSMKHSRLYHNICLVLFHWVPAMLVDALLYVLRYEPVLMRVQRRINKGFEVFEYYTNNQWDFKS

DKGQMVRKRLNEREAKEYKVDADGADIPQYFEHCVLAARRYILKESDDTIPAAKRHMRVMWVVDVIFRIV
MYGLIMWYLYKWTSSIFSSSSVEPAERMLEF
>FAR3
MSAEGFTAEEVDAMPDRIAATFTNRNVLITGGTGFMGKVLVEKLLRKCPDIGQIMLLVRQKKGKTPKQRL
EELFNDALFEKLLKLRGGIGALLSKMTLIAGDAGAPDLAISAADRQTIVDNVHIVIHAAATIRFDEELKK
AVMLNVRGTKLMLELAKECKNLLLFIHISTAYCHLHEKLLEEKPYPPPANPHQIIQAMEWMDEETVAALT
PKLLSKLPNSYAFTKALGEALVVEAMQHIPAAVLRPSIVIPIWQEPVPGWTDNINGPTGLLIGAGKGVIR
SMYCKSDSYADYLPVDVFINGIMICAWNYITRGDTKSNIVNFTSSAEIKVTWSEMIDAGREIIMNRVPLN
GVAWYPGGSMKHSRLYHNICMVLFHWIPAILVDILLFCLGYKPVLMRVQRRIGKGFEVFEYYTNNQWDFK
SDIAQTVRLSLNAKERRDYKVDAGGLDISKYFEDCIRAARIFILKEYDDTLPAARRHMKVMWCVDILTRC
LFWGLMLYWMSGWFSSLYSYILGSSEQSSIVNEMAA
>FAR4
MPEESEVRAFYTGKNFFITGGTGFVGLCLIEKILRSIPDVGKVYLLMRPKKGKEISQRLEEFPKNLVFEK
LLETNTTDVFKKLIPISGDVGQENLGLSAQDRQVLVDNVDVVIHSAATLDFQENLRPTVNINLLGTRRVM
ELCQQMKNLKVMVHVSSAYVNSYLTEAHEKVYEAPEDAEKVISLVGTLTDESLDQIEPKLLKTHPNTYTF
TKHLAEHEVVKCSDKFPCTIVRPTMIVAAWKEPVPGWTCSKVGPQGFMMGAGKGVVRRLPLAKENIADYI
PVDVVVNELLVAGWQAAKAKSGLSVYHCSSSTCRPFAWAMLENQVNGMLHKYPLKGAVWYPHLKFVPSLL
LFRISAIFVHFFPAILLDMLLRLTGGRPILFRLHKNVWSSLSRLERFIFTEWKFHNPNTIELARKLNQTD
RELFNIDISTLHWEEYFTKLLLGVRRYLNREEEKTLPAARSKDSMLLVFHIIWQILVIALLWYLVACVTG
LTMTQSAWAAPAIYILYTCL
>FAR5
MDPAQALEASELARHRVINDTIARGDSRIQRFYSGAVIFITGGSGFLGKQLIEKLFRACEIKKIFILLRS
KKQKTSQERLDELFKDPIYEHVHKVRPNFKEQIVPVDGDVMEKRLGINDEDWKMITEEVDIIYHGAATTN
FTDPLKKATLTNVHGTQQMLHLGKACKKLRCFVYISTAYTPATPERARKPVLEKFYDSPMSPDTLIQMAE
TVSEETMNAITPMFIKPWPNTYTFTKAIAEDVVRSVGADLPICIVRPAIVICALREPIPGWIDKSSVYGP
SGYVVGGGLGVLHVMYADKEICQDLVPVDLVNNAIISAAHDTVVRRERGDTDIRIYTVTSSRNPIKLRKV
IEEIYSAGMQQPTPFCIWYAFVRVTTNRLWWYILAWLLHYIPGYLVDGVLKLLGKKPLLMKVYQKVDKLA
AALEYFVTQEWFFQDTNTREMFWNQSPEDRTIFNCNMASLEWVSYLSLWCLGCRKYIVNDGLTGTLAGRR
KQFCFKIAHYSIIMLFLYLIFKLCSAFYGIVIR
>FAR6
MENIKIMSTGTMDSIQKTMEWNGNDPATPNFNETNQESLESIADFYDGAVVMVTGGTGFVGKALLEKLLR
SCPGIDTIYVLMRPKRGLTVEQRYKELLKNQVFDRIRARWPERLSKLIPIAGDVSAPNLGVSGDDMQLME
RVSMLLHSAATVRFTEPLHVAAALNVQGTAHMLALASTMPRLKALVHVSTAYSNAPKSHIEERVYPPPYD
PDSILRCAKMLPPETMEVISASLQGDHPNPYTLTKALAEAIVYNHSADLPVCIVRPSIVTAAHQEPFPGW
IDNIYGVTGLLMEMSRGTYRSGYCKQEMVIDIVPVDMVVNSCILAAWRQATKKPGRCPVYNVTSGSVNPL
QWGNFSRLCMKWSRENPTKYVMWYPNFTFTESRFMNTFWEVSLHFLPAFLYDMLLRAQGRKAIMMKLARR
FKMAAATGEYFANHEWQFGITELTALHNDASNTLDGSSFPHWPSIYDWNQYMGSYMMGIRKFILKDTAES
LPAARTKLRRLYWVHKLLQAATGYYIFKFVAGRLR
>FAR7
MASVFSGHYSGGQEYVPVAEFYADKSVFVTGGTGFMGKVLVEKLLRSCPKIKKIYLLMRSKRGQDVASRL
TELTQSPLFETLRKERPQELNKIVPIVGDITEPELGISPADQAMLCQKVSVVFHSAATVKFDEKLKLSVT
INMLGTQQLVQLCHRMLGLEALVHVSTAYCNCSRERVEEVVYAPPAQPEHVVTLVQTLPADLVDRITPDL
VGDRPNTYTFTKALAEDMLIKECGNLPVAIVRPSIVLSSLREPVQGWVDNWNGPNGIIAAVGKGIFRTML
GTGARVADLVPVDTVINLMIVCAWRTHLRRGDGVVVYNCCTGQQNPITWQRFVKTSFKYMRKHPFSEVVW

YPGGDITNNRLKHGILSLLQHRAPATVMDLVARASGKKPMMVRVQNKLEKAAACLEYFTTRQWAFSDDNV
QALCAALSPEDRRIFDFNVKNIDWDAYIESYVLGIRRFLFKESPDTLPKSRKVLSRLHIVHIITQVATVF
FLWRFLFSRSQALRSLWRRIVDLLSRAARLLAIA
>FAR8
MESTPIVKFYKGKSILITGATGFLGGVVLEKLLYSCPDVKTIYVLIKSKKGICGQERLTHLLNLTIFQRI
RNENPEVLKKVILVSGDMRDDNLGLSAEDMELLRKEVSIVFHIGASVNFAQPLLEIVNANTRASEKMLRL
AKSMEKIQAFVYVSTAYSNCRQNNDVIDEVIYNMTTSVQSGYYAVDNIPPTNNLLHNLIDRRPNSYTYSK
ALTELLIASEFNDLPVAVVRPTIITATMKEPIKGWVQGWNGGNTVILFQGIGLIRSWNCNVDAIIDIIPA
DIVANLIVATAWDIARNNTKRQGNIKVFNCASSSQNPLTVGQLFYTSLSHSKKIHSYLIPPYPLLIHNRT
LHSIIFFFLQILPVYIIKGLCGIFGRKPRIVQLFEKIPKLEISLPFFTSNQFTFIDKNTRQLYMEMNAKD
KRIFYFDVKIIDWYSYLETYVMGLKEYLLKL
>FAT1
MNETETLKPDILDSILEADDTDTNNTWEWYQEAIKTWLEDPGVEEHEGLNITALKMDQAYTVGLADQQVA
VYSVSDDCFRCPFELQKTLSADTEESWVVSTARRATWRVYTGIDDDYINSRNTTGMLCQLRPELGQFGVY
YLNITGESCEVTTTREPVDIYSPLLLLLGVLGAAWVLYGVGRLIMSRVRRTGKLRYGDKELAIQQRLRAL
DTFRGLAIVMMIFVNDGAGGYWWLEHATWNGLCAGDLVFPAFLWIMGVCVPLSVKSAFAKGVPRWKICAH
ILRRSVIMFLLGMSLNSIYGSNMLHELRIFGVLQRLAVAYLVSAGFYALTAPKYYTPPRGACGQALKDVL
SCVWCWVLAAGLITVHTVITFAVHDPHCPAGYLGPGGKHDDWVAPNCSGGAAGYIDRLLLGDSHLFQRSD
ARRVYGGLATDPEGLLGCLTSSVQALLGVQAGATVLLQRSHKSRVSRWLAWALVFALVGALLAGFSKEHG
SIPINKNLWSMSFVLVTSACSLVLLSICYVFTDAWRLWGGGPFRAPGLNAIALYIGHSLCSHLFPFHWKV
PHMRTHSIHLAEAVWGTALWVIIAHVMAKKKVFITL
>FAT2
MSLGKFENVGAFVRDCRNGTLLYDEACLEVITSETVTFWSQYEECEGCKLVLTKTLETTGEIVLKTLSPV
HYAVKNGNGQICNGTYLFGEFGQYTLNLTEATKEECLPRMKAEPDAAHLPILTAVVVLLAMATLWYIVKG
IGKRLLTHSFVANYFNREDNELGSETRVLTAEVSAPRAPTRSRLRSLDIFRGFCIALMIFVNAGGGGYAI
FSHSTWNGITVADVVFPWFAFAMGEALVLSLNARLRTSLPRTTAFYQVARRALLLAIIGIILGAVNVSWA
HVRLPGVLQRLAAMYLVVGALECVFMPTSQNITPGRSLFRDIAAGWRQWLATIVLVTVQVCVTMLVAAPN
CPRGYVGPGGLHMSAYGQDLRGCTGGIAGYIDRVVLGASHLYKRGSFRILYAATVAFDPEGLLGILSGVL
VVQAGAHATRIMLAYNHARARIMRWVFWSIIFGVTGGALCLFSKNGGPIPINKNLWSLSYCLVTSSMALF
IQAVLYFIVDLKNKWGGRPLYYAGQNALFLYIGSELLKKHFPFYYSVPAPTHAQLLATHAATMLLWLAVG
VALYKRRIFITL
>FAT3
MTAFNVVLGCAMALFIILLTISSITRYYVKFTLFVVLSLIFATAPIPLMLIKPFDPKNALIPAALLRVTS
RMLGMRWTVRGLENVDNSRGAVILLNHQSGLDLYVLAVLWPLMERCTVVSKRSLQYLVPFGTAAWLWGTV
YIDRGAQSARDALNKQTTAVREHKRKLLLFPEGTRHSGDKLLPFRKGAFHVAMDARAPIQPVVVSKYHFL
DSKRRRFGSGEVIVTIMPLVETEGVNKENIGELIDKVQADMQETFSRTSAETLPVRMKAE
>FAT4
MISFITKNKNDIIYLSLLLFSVAVGPYYRSINSIQIKKWVGSLLGLLLIVIVSGYCAFHPISSAIVGIIL
IKLATVKYCHIVTFFFMFGYLFFFRLADKFGLPLSSGQTNLIQMIIVLRVVGVAFEINGSWRAVGAGKKK
EDKPDNDKKEKDADFLEIHNPSLMDLFHYSFNYVALLTGPYYRYRTFDDYFNLPYSKYADCAGFTINTLK
TVPLYISLYLAMSNVWPLEYILTEDHNNRSFLYRMLYPWVLFAAFRQRIYSGMTLAESVCTSAGVGAYPV
EGKNRTGHGPTVGYLKLKQMTEEEAKRAQYDFNTVESMDVWGCETVVTLRDSMKVWNKAVQYWVAMVVYK
RFPVKSLKIHAALFVSVIWHGYHAGYFFCIYFCPFYLMAEDIYYKLYYKDATGTKKKIIGFIMWFLRSHS
ESYQAAAFLLLTFDRIWIYYSSVYHYWYGCWLAFLIIGLILNQIHKMGRPKKLKGDEKQPISSNH

>FAT5
MEAMVAVWTMVEHVRVASGAAELAIFTALLYWFFTSSRVADMLEVVGERVTGWCGRDSSGSLRQAVRARR
RPRHDVYAKVDAERTTRTTSLYRVKEGPVVPAPQPVTRPPAGLACARCAPLSRESWQDPKTPDTGSVINI
LDIGRQTFANGGVVSRYLCDLAQCLNLIKYDYKDIVPEVVKDERFQKAIEETTQEELRKAAQAEEGGGEA
GSAARYPAVRQRVQARAMRVLRDISSAMSNNVLKLVAWLCHKAIRRVAGGGCGTRSACIERLRRAKAAGL
PLVFVPLHRSHFDYILVTFTLYLTGLRPPLVAAGDNMRIPFFGWFLRGCGAFYIRRRVDGSEYHGDPVYK
SALRAYILNSLAANNNLEFFIEGGRTRTGKPQPPKAGILSVILEAYLEGTIDDALLVPVTLNYDRLVDGS
FVREQLGMPKQMFWSALRGIWRTLNTNHGSIRVDFNQPISLKELVTSFQKYNYLKAPIERTLTPPNNNLA
VNLDRQILYNHSHSSLYGADVSTDHKMMVEAIGRHLVYDAAQATALMCTNVVSYVLLTEERAGCGVRQLG
ARVAARAAALSGAGRDLYAGDAGTVLKHALDMLGRGLVRREGSGDRQQVRGVLSIAGALELSYYANAVVA
HYAAPAILATALESILTQPDADQEIVRHSELMEAALQLCNVVSQEFILCPPCHRIEERMLDAVDTLINDR
VIERVQEEEGIAEQQWARRFAKTLELDDDDEMPDPTRNIKYKVCKQPEALAERRRLLLTIRPLLEAYAAT
CRCLDSGSQKELEKRILNTLTEDFTQNKMVYGEAVSTDAIRNCLRLLRQWGVIDMYVENRERRMRLRTPY
DKQAARDYICNNIYKFNMTTSLLH
>FAT6
MWGFFLGVLGWVGLSPVPFLANLIGTTEPAFKLLISIIMGYPIAIVYHKIIRKKYDGYHNIYFIITGLDM
AYYNFGYTLYHNLIPALVMYGTTKVLGPGKENAIVTFAFNMGYLLIGYALTESEEYDITWTMPHCVLTLK
LIALAFDLWDGAKMLKGEELSENNKKTALEKSPTLTELIGFIYFPACFLVGPIFSFKRYTDFINGKFPLE
KDESVYESQAITRLVQGMLYLIAFQVGVSVFSMQYMLSDEFLENSIFYRHIYCGLWAHFALYKYISCWLL
TEASCIRFGLSYNGVAKTPEGQLESKWDGCNNIKLLRFESATKFQHYIDSFNCNTNHFAAEYIYKRLKFL
GNRNLSQFFTLFFLALWHGTRSGYYMTFLNEFLIIFMEKGLESILTKTELYHKMWSNNVAKYVLYAILKT
YTIVFMGWSLVPFDVKVFSKWWSIYASLYFSGYLLFVPWGFLYKPMMIKLAKYLSREKVQ
>FAT7
MAGWYDYPTLEPYKLDRHLPLNHAWVIANTSISDVDIYSRREDCYECQYELHKSLKSNKPASWMVDSSSQ
VTWLVMRSKQGRPTSDMTKKSWLCEMRPTFHEGGIYRLDVGDCEIKPEIPVPWTSLSLLASILILLVTCT
IYTFARFMYNRKQQREKASHGKALEASNENDEQNPALNVFKGILMSMIIFVTTGGGGYAFLTTPKSGITL
AEGLLYMYVWSIGVNIPTFMDKAFRENTKCHIFRDIFVRALILVFMGTTIDSIVAYQMFSISLWCPLQKI
GIAYLIVATAYVLTVQRFEMKSKKIICKAIKNLISLLWIWIIAGFLIAIFIILTSTFVSFNCLSNTSLQY
KRPYFWTPPPPETQNCNNHVSEYANKILVGQSKKTVDVHRFIAFTGGAMGILSTILIMMIGLKSGVVYLQ
HQAHSDRLKLWGLWATLFGLGGTCFAVVTYPLEFNLWPLSSIQLIGVAAMCIFSLIYVITEVWRFNSYIL
SSLGISSLVVLCGHVLFSGSIPFRWGIVLDNHVMFLLQNYWIILLWIVIGIALRRRNVHFEI
>FAT8
HHSDLYQLIFEAVRSGEVSEIERLVEKLGSEVLSARDQHGYTPAHWAALDGSVAVMRYLVERGAPIDLSC
LGTQGPRPIHWACRKGHASVVQVLLQSGVAVNAADFKGLTPLMTACMYGKTATAAYLLGMGAATRLSDIN
GDTALHWAAYKGHADLVRLLIYSGVPLHCTDNFGSTPLHLACLSGNLTCVRLLCEKVKAELEPRDKNGKT
PLMLAQSHRHAEVVKLLQKEMKRKSHWMPPLSELWALLFGGAGDSKGPLLFFLVSVLLWGYPMYVIRCIP
LTWNTLRLSHYCFLYWNAIMWISWVIANRRDPGYIPQNSETYYRAIRQIPYYDKWKKRNVILSRLCHTCR
CLRPLRAKHCRICKRCVAYFDHHCPFIYNCVGLRNRMWFFLFVMSVAINCTLSIYFACYCLLLEGFGLLY
VLGLLEAITFCALGWILTCTSILHACMNLTTNEMFNYKRYPYLRDKRGRYQNPFSRGPIMNLFEFFVCLP
DKCDEQDIFHEETI
>FAT9
MLEVVWKIVNGIWSILVGISESLGIQWAPVNLPMERRLQTLGALGWICLVLFGEILCIFIFLKIVYSDYW
WVGVLYAIWMVNDINICNEGGRKIELFRNLSWWRRYRDYFPLKLVKTADLDPSRNYLFACFPHGIVCAGA
FGAFATNALNFYKIFPGMSCHMITLGGHFLVPLFRDFILGLGGCSASQQSLMYLLDKRRHQGKCVALIVG

GAAEALDSHPGEYKILLSRRKGFVRIAMKSGAPLVPVFSFGEPDVFRPFQNPENSRLRRFQEAFRKYTGV
SPMFPIGRGLFQYTFGILPLRNPVTTVVGEPMEVQKNLEPTEEEVNAVHAQFTERLVQLFEKEKSKYLKD
HENTRLVLT
>FAT10
MAILRYVWIVARTTFAVVNNLYCIPAYVVWMMALRLVRPFFAPVYWKIEGLMYHWLLAMVSMWSWTAGYE
IVETGDEPVTCSGRRTLVLANHQSTADVPMLMAAWNPRPGVLPNLMWIMDRVFKFTNFGIVSVLHEDFFI
QAGKAKREASLEELKQHIYKFYVPLGRQFMVLFPEGGFLHKRREVSQRFAEKNGLPKLEYVSLPRAGAMK
VIMEQIGPHRDASGAGTSRQARKDNNFNQNNSKMILDTNSGDSIEWVLDVTIAYPDRIPLHLVDVVCGIR
RPCTTHLHYRLYPASEVPSDSEGMTQWLYDRFIEKDKMLEEYYRTGKFPTKSDKPHVLRYVRQDNLRYLI
LHTFFIASTFLQYKIFFYFYNNLW
>FAT11
MTSQFTFHSFRPFINQSVVKMALVMPLVSVAISILYTPFLLLILCVIFLASIGKSLGVRRLYVNILLKLF
EYGRQHIEVAKKLQRNDSSDEEEEPPAIPDDKPPSAIIKENGSLNGTKMTVIEREEILGPSPELNYKRSS
SQERTQNHENSHHTSLEFHFNHCLDLVKAGMESIIEDQVTSVFEAEELRSWNLLTRTNRQYEFLTWRLTI
IWMMGFIVRYFFLLPLRICIFFVGAITLIVSTAVVGMLPPCKLRRYLGQFCYRTSFRTMTRGLSCVINYH
DTQYMPKASGVCAANHTSVIDVGILSCNATYSLIGQRHNGFLGLLQRALSRASPHIWFERSEVKDRHIVA
QRLKEHISVPDNPPILIFPEGTCINNTSVMQFKKGSFEVGGTIYPVAIKYDPRFGDAFWNSARYGMLHYL
LNMMSSWAIVCDVWYLPAMRREHGESAVDFANRVKAIIARRGGLVDLMWDGQLKRMKAKKEWRELQQEEF
SKRLKGE
>FAT12
MSQNVRQRRPQPQQQTQIQNGDVKHGSNEGQDQEFVVRESPLTVLIDTSLHIKAIYHIFVVILLVMFCDT
VIYDLVESGKVNVGLSTVKHGFGDIRRGAKLWLFEFAIAMLFYPGMLVYSAGAKLFKKIPGLNVIWTGIG
LAGVVALEVAILAVPTWDLGRTHLALGSSVAVTTEMFRLAMKICAVAVACAPRYHGDSTPLPTLSQYVYF
LFAPTFVYRDQYPRTKKIRWGVVVFHLLEVAAIIFYDSFLWERFILPYWSDYGKQPKIEAGTVVRGMFAC
VLPGVISFLCGFYMLLHAWLNACAELLTFGDRLFYEDWWTKSQFSQYYRAWNRVVHCWLRDHIYRPLAPR
TGRACATFAVFLVSAAAHEMILSLSFGYFYPVLLMEFGVVGMLMVPLTATIGRRFPSCFNLLMWLSFFIG
NGILWSLYPMEYFARKNCPLSDTDSYFVPKSWSCPEIVLKPNWTFQNPF
>FAT13
MWYNIGLFAIVCLLTYFLKKIFSSEPNNVKFQFQFFIFYFLTSVVAALIWPLFLLNPKDVRNAKKAASIL
KHITKIYDLRWELRNGHILEEERGAIVVSNHQSTIDILGLFNIWELVDKMTVIAKKEIFYVWPFGLSAYL
AGVVFINRRNAKDAYNQLKETSDVMIKKKTKIWVFPEGTRNKDYTKLLPFKKGAFSMAVSSQMPIIPVLF
SPYYFINRKKHIFSNGHVVINCLEPVPTTGLTLDDVPELMSRVYDLMQAEYMKLSKEVLEALPKDYPHTS
VG
>FAT14
MSYYDYYDGSKLFLFVSHRVGLPIDLVNFLIAQIAALCLARLFRKPLRFASSEFRHSVCLIIGLIMGYFC
FGKQAVHLSVLPMLTYTLLKTVPNHLTGNVILAASMIYLSCLHMHRQIYHTADYTLDITGPLMVITQRVT
SLAYSLQDTLSKKGADLNMASFSDSSKLSHIEKIPSPLEYFAYTLAFQTLMCGPVVFYADYINFIEGARV
DGDEKRMNGAAEPSPRLAVMYKVAGSLSAAMLYLTLAKKYPLAVLAELTDPASEVSRSWSAVYLLWYAYL
STLVVRCKYYHAWLLSEAICNNSGMGFNGYDTNGKPKWDKMSNIDVFGFEFAQNFRTAVACWNKNTNAWL
RYVAYERGSKAWRTARVYALSAVWHGFYPGYYLTFFAGGIFTVAARKIRGVARPMFLGSRSKKLFYDFLS
VVTTRVAMTYATVPFVVLHLAPSLAFYGKLYYSLHFIALGAMLLPDKSTHSSTSSSVSSELPSKSSKLSV
QTNGDIKVS
>Gilat_FAT15
MFYNLKCVVFCFYIIYIMSKFYILSFRFASQIVKHVTKIIEVKWSLRNGKYLAEDRGAVVVSNHQSSIDI

LGMFNIWHVADKVAAIARKEIFYVWPFGLAAYLAGVVFIDRNNSKDAYKQLKVTSEVMIKNKTKIWLFPE
GTRNKDFTKLLPFKKGAFNIAVAAQVPIIPVVFSPYYFINRNKYIFNKGHAIIQCLEPIPTEGLTMDDVP
ALIERVRGIMDAAYKELSKEVLSALPNNYPLCTTD
>Shemer_FAT15
MMWDKIIYIFMIIITTYIVKQLFSETPNFIKFKSKFLVFYIWTSLTALILLPFFVFNPKNVKNSLFASQI
VKHVTKIIEVKWSLRNGKYLAEDRGAVVVSNHQSSIDILGMFNIWHVADKVAAIARKEIFYVWPFGLAAY
LAGVVFIDRNNSKDAYKQLKVTSEVMIKNKTKIWLFPEGTRNKDFTKLLPFKKGAFNIAVAAQVPIIPVV
FSPYYFINRNKYIFNKGHAIIQCLEPIPTEGLTMDDVPALIERVRGIMDAAYKELSKEVLSALPNNYPLC
TTD
>FAT16
ALVMPLVSVAISILYTPFLLLILCVIFLASIGKSLGVRRLYVNILLKLFEYGRQHIEVAKKLQRNDSSDE
EEEPPAIPDDKPPSAIIKENGSLNGTKMTVIEREEILGPSPELNYKRSSSQERTQNHENSHHTSLEFHFN
HCLDLVKAGMESIIEDQVTSVFEAEELRSWNLLTRTNRQYEFLTWRLTIIWMMGFIVRYFFLLPLRICIF
FVGVWWLLICTACVGTLPDGVTKQRVNYAVSIMCFNFLSRCLSMVITYHDTHNKPKNGICVANHTSPIDA
LVLMCDNCYSLVSGQCSPTACIAPSESPPGSNLAWPTWKSLNRLRTQVGRCKDN
>Gilat_FAT17
RKPMTGWRRALKPLICFIGKLSYLAGGMAITIRGRQASRQEAPILVVAPHSSFLDSCIVYATKMSSVIVR
KESMDNYVGKLINYTQPVYVWRDDPNSRQNTIKEIIERATSKEDWPQVLIFPEGTCTNRSCLITFKPGGF
YPGVPVQPVTIRYPNARDTVTWTWEGPGALKLLWLTLTQIHSSCEIEFLPVYYPSEEEKRDPKLYARNVR
DVMAKALGVPVLDYTYDDCRLIARAKQLGIPAAPSVREVSELRTHLGLERSQLELHVAAQGMSGRPRWVT
SAEFFARLGISTADPHAQRLFDIFVQRSTGLACFPDYLLCAAFLSLQHAPLSQLLSYAFKLYDSTGTGRL
TATHFEEVATRCLGLCQEDAQNTFRQADLDEKGYLTYDEFISYAQKKAEFCHIFATDAAHQKPKTQ
>Shemer_FAT17
MNGKMHSETGAFSSDGMRAEILNPFVHKLELDNTYDKIKTAIFTVILLPFRVIVICYLIVTAWLIACIGL
YGLSEEDLRRKPMTGWRSKLRFSILSLMRLVVVAAGFHRVRILGHHHKPAHPRDAPVVVMAPHSSFFDAI
AIVVLGAPSVVAKADTARLPFIGQLINYTQPVYVWRDDPNSRQNTIKEIIERATSKEDWPQVLIFPEGTC
TNRSCLITFKPGGFYPGVPVQPVTIRYPNARDTVTWTWEGPGALKLLWLTLTQIHSSCEIEFLPVYYPSE
EEKRDPKLYARNVRDVMAKALGVPVLDYTYDDCRLIARAKQLGIPAAPSVREVSELRTHLGLERSQLELH
VAAQGMSGRPRWVTSAEFFARLGISTADPHAQRLFDIFVQRSTGLACFPDYLLCAAFLSLQHAPLSQLLS
YAFKLYDSTGTGRLTATHFEEVATRCLGLCQEDAQNTFRQADLDEKGYLTYDEFISYAQKKAEFCHIFAT
DAAHQKPKTQ
>DHr
MFTLGNETNITDNFSMSAVDPTDIFGPQRDSLYIVLPITIIYTLIFITGLLGNIFTCIVIIRNKSMHTAT
NYYLFSLAISDLLLLVSGMPQEMYSIWSKWPYVFGHTFCLIRGLAAETSTNASVLTITLFTVERYLAICH
PFVSHKMSKLSRAMKHVMLLWVVSFGLALPQALQFGIKDHQGVTMCLQTRVIIAHSFEISTFFFFFAPMI
LITVLYSLIGLRLKKTSISKQTKEKEDFERNMRFNHRIRRKHSQSTRRVVKMLVAVVVAFFICWAPFHAQ
RLVAIYGTAENHLARSPVLLFVYSTLTYISGIFYYMSTCINPIFYHIMSNKFREAFKNTMMHWCCREERR
TLQRCSYTAIAFARHPNSNGTINSGNSTRNETPIHTKATRLDSKEQIINIDGQQFRVCDYCNRAMPTAGQ
MCRTHGCHHKYTVDFKNKRDHVDSNPRSDRSKPNFIEMSLVPEKRKFCGPSCCAAQESPSETPTPPTPDA
ATGSSEENRTQYYPVQSDKYTKEIKLRMKQR
>ETHr
MISISNIAQTESTLHNFTYKHVASNYNTSFNNAVLFDNGTRFDVSNHSYGSENFTEYAEIPYYIKATSMT
FCIVIMCLGVIGNVMVPIVIFKTKDMRNSTNIFLVNLSIADLMVLLVCTPTVLVEVNSKPETWVLGKELC
LAVPFVELTVAHASVLTILAISFERYYAICEPLRAGYVCTKTRATLICALAWFFAALFTSPILAIAEHHE

NRHDGSISFQCLTQAVTFWQITFFVMVIILLYVLPLIILIILYSVIAKNLITAASKVVMNKTVDPYNARA
RKQVILMLGTVVLCFFLCLMPYRVLALWIIVTPQEISEEISPEKWYNILYFSRIMLYINSAINPILYNLM
SSKFRIGFCKICVCYKRSSDRIQNRAQRTITNGSTTSSSLTRTTNSLKKLFTHRGSLDKSDTESENRESK
DETKPNGFFERIFTNKRFIRQQSAPVCANVKPKINRMRSEGNVFEVTDLDKGTENDTVKFIGHSNSGKTN
TDFDLDRNRGSLRRSVLINSAKAKSVEDRKKISYQKIKVESVVGYQNTKSVDYDFPESFV
>Octo_r1
MDQVNISTGNETGNSTNVEDGWTHDIMFKIRISVLLLIVIMAVLGNMLVIVSVMRHRKLRVITNYFVVSL
AFADILVAVLVMTFNFSVQFYNKWIFGPIVCDLWNSSDVYFTSTSILHLCCISVDRYYAIVKPLKYPIKM
TKKMAFVMLAATWLSPITISYAPIFMGWYTTNDHLIQRDKNPDICDFIVNKPYAVISSSISFWIPCTIMI
FTYLAIFKEANRQEKALHARAGNAMLMHRHSREVSDKNGALHINANTPTKDKNILKMKREHKAARTLGII
MGAFILCWLPFFLFYVSTSLCDSCWYPDVITVIMFWTGYFNSALNPIIYAYFNRDFRNAFKNTLACAFCS
FCKRSASDLDALDRLDRRGSAQLRVPIPSRRASDLASL
>Octo_r2
MRSLNETACAALLEDVRWDEPTSLVSLAILALIDVLVIAGNCLVIAAVLCSSKLRSVTNLFIVSLAVADL
LVGVAVLPFSATREVFEIWIFGDVWCAVWLAVDVWMCTASILNLCAISLDRYVAVTRPVSYPSIMSRKRA
KALIAGLWVLSFVICFPPLVGWKDKKPDESEVKDDVPPNPRCPWTCELTNDAGYVVYSALGSFYIPMFVM
LFFYWRIYKAAVRTTKAINQGFRTTKGGCGKGMGSRFDDNRLTLRIHRGRGSARPHGSPLSTASNHSTST
SLSASPERLRRHSSARRAHEKIKISVSYPSSEQICPAHENSRSPSRSPSPSLYAVHYERDGRELTESRLR
VRPPHHLAPTPLYDDFDDKPRTTRRMGKRNIKAQVKRFRMETKAAKTLGIIVGGFVFCWLPFFSVYVVRA
FCGDCVTPIVFSVLFWLGYCNSAINPLIYALFSKDFRFAFKRIICKCFCGGGGAGARRESDGANAANAVE
SRARPLRNTHSHSLEEHDHSQSHTSSTTDR
>SPr1
MADKDWADYYGVQYNNSRPNIKPGLYESYDAENDVIDVVGKENYSFYDNQTFPPYSNFTTDDFCANYSHV
YINVTCEFAINYAEPMYGYIAPFLLATTIVANTLIVVVLSRRHMRTPTNVVLMAMALCDMFTMLFPAPWL
FYMYTFGNHYKPLGPVQACQAFNYMNDVIPAMFHTASIWLTLALAVQRYIYVCHAPVARTWCTMPRVNKC
LIYIGIAAFLHQLPRFVDREYVPHTTVWRGHFEDVCRMQMAPWVHSISLDAYFITYFGFRVLFVHLIPCT
SLVVLNVLLFRAMRAAQLNRQKLFKENRKSECKRLRDSNCTTLMLIVVVTVFLMVEIPVAVVTLLHIISS
TMVEILDYQIANLLILVTNFFIIVSYPINFAIYCGMSRQFRETFKELFIRGTVTSRKNGGSSRYSLVNGP
RTCTNETVL
>SPr2
YIAIKYPSHSPALCTDRRCSLAILSSFVLPPILCIPSYFVFTIHKEFTVDRRKVYFVDSYYEGSLYQINF
WVHAVLIKLLPCFILTVISAWLIRALYRANSRKKALKGYSACHTDTVVNGNGNVFTRKSTKRSKAERRTD
RTTRMLLAVLLLFLLTEFPQGILGLLSGVLGRCFFKRCYDLFGELMDALALLNGAINFVLYCSMSRQFRM
TFGQLMWRGHLYRWPPPTASHSDGQNTAKSSIP
>SPr3
MANATDAYCVPGATDFNRAYSRLHGYIAIVICIVGSATNSINIAVLSRREMASPTNSILTGLAVADLLVM
LEYIPFALHMNIKIGPQVNKNTYAWAVFVYFHSIFSQTFHTISIWLAVTLAVWRYVAIAYPQKNRSWCSK
RNTTLAIVSAYVICPFLCLPIYFAMTIVPSEVTPQNNSEFAEDLSLPHNRTVYVLEMSKNVELVTAIMWI
YSVILKLVPSIALSILSTCLISKLTTTERRRQKLLKRSTVGPNEAEKQCLAEESCARRSSRTDRTTRMLL
AVLGLFLSTEVPQGLLGLASALAPDFFKSCYGMFGDLMDVLALFTSSVNFVLYCSMSRQFRCTFARLARR
MLSGAEEPAKFAAKLEPTTQVSFFNKGSPGRYSDRSPP
>SPr4
MTNQTRTLLEFFKILAYGNSTDGAFNKTQIKSVLRAQAEKGSGDFESLLVRVMADAKSELNISIKPDTCG
YCEGDFRDVVQAYNRIHGYVSLLICTIGVIANTMNVAVLTRRDMAAAPINRLLKWLAVADVFVMLEYVPF

AIYKYLVLPEKLDFPYSWAMYLLFHMHFAQILHTASICLTLSLAIWRYIAIKYSDRSHILCTERRCSIAI
LTSFILPPILCTPTFMVFDIHMKKLDEAGVDVAYHVDADYQGTLYQATFWVHAVLMKLLPCSILTVISVW
LIRALYSANQHQKNLRNYSACPAAEKMVKRQHKADKRTDRTTKMLLAVLLLFLVTELPQGILGLMSGLLG
WCFFKRCYDLFGELMDFLALLNGAINFILYCTMSRQFRQTFRQMLLQPHLARFMPPHASHSDSQNQNTAK
TSIP
>Shemer_SPr5
MLADDENRRVYNDTYTNVSYTLFEDSRLISLLKYGGPNNTNIGEVLRALEDYRNKYNISVMKECHHSEYC
SGEFRDLILAYNNIHGYVSLLVCLFGSLANALNVAVLTRRDLAAAPINRLLKWLAVADVFVMLEYVPFAI
YKYLVLPEKLDFPYSWAMYLLFHMHFAQILHTASICLTLSLAIWRYIAIKYSDRSHILCTERRCSIAILT
SFILPPILCTPTFMVFDIHMKKLDEAGVDVAYHVDADYQGTLYQATFWVHAVLMKLLPCSILTVISVWLI
RALYSANQHQKNLRNYSACPAAEKMVKRQHKADKRTDRTTKMLLAVLLLFLVTELPQGILGLMSGLLGWC
FFKRCYDLFGELMDFLALLNGAINFILYCTMSRQFRQTFRQMLLQPHLARFMPPHPSHSDSQNQNTCTEK
VKR
>PBANr1
MELDYDDLAANVSNVTVKAAAYREPESLDLLVPLTVTYSIIFVAGILGNTSTCVVIARNRSMHTATNFYL
FSLAISDLILLVCGLPIELHRLWIPTTYPFGEAHCIAMGLASETSANATVLTITAFTVERYIAICRPFMS
HTMSKLSRAVRFIVAIWVCALCTAVPQAMQFGIVSYKENGQNVSACTVKGHGVHQVFVVSSFVFFVVPMS
VISVLYALIGIKLRTSRVLHPAKKLSVESNDRSNRSVRYRNGASQRRVIRMLVAVALSFFVCWAPFHVQR
LLAIYGKSLEHPSDMFYQVYIVLTYLSGVLYYLSTAINPFLYNIMSNKFRNAFKVTLSSWCGRKSVPGLG
RTYSALLASQRARVPGGAVSERVRGPRRLCRLATATTQLYDAPARAQVSV
>PBANr2
MELDYDDLAANVSNVTVKAAAYREPESLDLLVPLTVTYSIIFVAGILGNTSTCVVIARNRSMHTATNFYL
FSLAISDLILLVCGLPIELHRLWIPTTYPFGEAHCIAMGLASETSANATVLTITAFTVERYIAICRPFMS
HTMSKLSRAVRFIVAIWVCALCTAVPQAMQFGIVSYKENGQNVSACTVKGHGVHQVFVVSSFVFFVVPMS
VISVLYALIGIKLRTSRVLHPAKKLSVESNDRSNRSVRYRNGASQRRVIRMLVAVALSFFVCWAPFHVQR
LLAIYGKSLEHPSDMFYQVYIVLTYLSGVLYYLSTAINPFLYNIMSNKFRNAFKVTLSSWCGRKSVPGLG
RTYSALLASQRARVPGGAVSERVRGPRRLCRLATATTQLYDAPARAQCMNGRDLSTVSESPNGNGHWTHA
WKLRMNEPSDSIGSPRSISNSSLREVDEELTGEELATYMYQVNCDIGGLT
>CSP1
MSSCSKTLILLSIMLLVNISFCKQRNYDNFDIENMLSNPMRSKRFFECIRNESSCTTEDKEMKHDLVEMI
TTSCAGCTDIEKSKLQSLKRRAMDDPQILNLALGDM
>CSP2
MKGFIVLACLVASVLAADKYNPKYDNFDVETLITNDRLLKSYINCFLEKGRCTPEGSDFRKALPEAIETT
CAKCTDKQKTNIRKVIKAIQQKHPKHWDELVAKNDPTGAHRANFDKFIQGS
>CSP3
MKTFIVCVLALAVIAAARPNEEYTDRYDNINVEEITGNKRLLIPYIKCVLDQGKCSPEGKELKSHIREAL
ENYCAKCTEVQKTKTRQVLRHLINNEADYWSQLTAKYDPQRKYTQKYEDELKTLKA
>CSP4
MMSMRYLVLLCCVVAAVVADDKYTDKYDNINVKEILENKRLLQAYVDCILDKGKCTNEGKELKDHLKEAL
ETGCEKCTESQKNGTHTVIQHLIKHEIEIWKELCAKFDPSGTYRKKYEAIAKEHGIEIPE
>CSP5
MKLFIGLCLMAMAAAQTYSPENDNLDIEAVVNNDDTLKAFYGCFMETGSCDAVQADFKKDLPEAVEQACA
KCTSAQKHILKRFLEALKQKFPQEYEEFRKKFDPETKYFTKLETAVANS
>CSP6

MKTILVLCAVLAMALARPDGETYNPAYDSFNAQELVDNERLLKNYGKCFLDKGPCTPEGTDFKKRIPEAL
RTNCAKCTPKQKELVRTVVRGFQQKLPEVWQELAQKEDPKGEFKEAFEKFLSGKD
>CSP7
MKFFLVTLAFTVSCVMAEYVGQEKYTDKYDSLDLDEMMSNKRLMQAYCRCILDQGKCTAEGRELKSHISE
ALQNGCAKCTDRQRYGMRRVIGYVMKNENDMWQQMVQKYDPRRIYTTKYE
>CSP8
MNWLTLLVGLVVVALAVCEERYTDRYDEINIDEILSNRRLLLPYIKCLLDQGRCTPEGKALKTHVVDGLQ
SACSKCTEVQKKMARKTVKHIREHENDSWEKLKAKYDPGDKYQEVYEAFLAANDE
>CSP9
MKVLMLCFQLFMLAVAQEYYDRRYDYFDVDSLIQNPRLLRKYLNCFLDKGPCPPIGIVFKTLLPEVVMTS
CAKCSPRQKRFARRVVAAFDRYMPQEHQQLKMKLDPQNKYYDNFVKAINSS
>OBP1
MVLLIVLVKFVVLLTLCEAMTMKQIKNTGKMMRKTCMPKNNVEEEKVDKLGQGIFIEEKEVMCYMACIMK
MANTMKNGKLSYDAAMKQADLLLPEEIKEPAKAALTACKKVPDAYKDVCEASFHVTKCIYKENPDIFYFP
>OBP2
MSSATTLPLLFMLLTVGHGAKEKPEFSEEIREIIQHVHNTCVDKTGAAEEDITNCENGIFKEDQKLKCYM
FCLLEEASLADEDGIVDYDMLVSLIPEQYYDRVTKMIFGCKHLDTPDKDKCQRAFDVHKCSYDKDPSFYF
LF
>OBP3
DNDIRGLEHHSCVICGLQWCCKMIELFAFFAFGVAVCAADAPVSPPVPPPVLCGEIPRSIFSCLGIPTVI
AASAGYQCRLHASECDKMKCIFEKSGWMDGDKVNKQKVIDHFNKFANDHPAWSTAVEQVKTACLSGTLPA
QGVYLDCPAYDVMNCALASFIKSARPSQWASIPECKYPQDFARACPICPNDCFAPQVPYGSCNACVLLPR
TP
>OBP4
MIMNQKELCMLVVVILACSTDAMTRQQLKNSSKMLKKNCMAKIGVAEDLIGNIEKGQFIEERDVMCYIAC
IYQMTQVVKNNKLNYEASLKQVDIMYPPELKDSAKAAIEKCKDISKKYKDICEASYWSAKCMYEYDPKSF
IFA
>OBP5
MISVLKLFLCVCFIGVCYAKSEAEIRKWFIGLAIECNNENPITPEDMTLMHEHKIPDNENAKCLLACVYR
KANWINDKGMFDIEAANAMVEKEHTDEPAKIDNSKKLFQLCKKVDDEPVTDGEKGCQRAALLTTCMTENA
PKFGIPLIM
>OBP6
MKAFVFLCMVVAAAAAGGHKAELSQSQKNTIRQYTADCVKETGVKPEVLANAKKGQFADDEALKKFTLCF
FQKTGILSKDAKLNTDVALSKLPAGVDRAAVGKVLEECKKKTGKTHADTAFEVFKCYHKATPVHLI
>OBP7
MYKIVAFCAFVFLGCLAAKTQKVQLAPEKAGKIIQDALQCVSETGADPAILIQIKDKKYSDDETVKNFFY
CAFKKTGMATKKGVIKVDKLLDIYPDTVDKEAIKKVILECQQENGEANERVYKFFKCYQENTPVRLSLMK
N
